# Spatial epi-proteomics enabled by histone post-translational modification analysis from low-abundance clinical samples

**DOI:** 10.1186/s13148-021-01120-7

**Published:** 2021-07-28

**Authors:** Roberta Noberini, Evelyn Oliva Savoia, Stefania Brandini, Francesco Greco, Francesca Marra, Giovanni Bertalot, Giancarlo Pruneri, Liam A. McDonnell, Tiziana Bonaldi

**Affiliations:** 1 Department of Experimental Oncology, IEO, European Institute of Oncology IRCCS, Milan, Italy; 2 Institute of Life Sciences, Sant’Anna School of Advanced Studies, 56127 Pisa, Italy; 3 Fondazione Pisana Per La Scienza ONLUS, 56107 San Giuliano Terme, PI Italy; 4 Department of Pathology, Fondazione IRCCS-Istituto Nazionale Tumori, Milan, Italy

**Keywords:** Epigenetics, Histone post-translational modifications, MALDI-MSI, Mass spectrometry, Proteomics, Laser microdissection

## Abstract

**Background:**

Increasing evidence linking epigenetic mechanisms and different diseases, including cancer, has prompted in the last 15 years the investigation of histone post-translational modifications (PTMs) in clinical samples. Methods allowing the isolation of histones from patient samples followed by the accurate and comprehensive quantification of their PTMs by mass spectrometry (MS) have been developed. However, the applicability of these methods is limited by the requirement for substantial amounts of material.

**Results:**

To address this issue, in this study we streamlined the protein extraction procedure from low-amount clinical samples and tested and implemented different in-gel digestion strategies, obtaining a protocol that allows the MS-based analysis of the most common histone PTMs from laser microdissected tissue areas containing as low as 1000 cells, an amount approximately 500 times lower than what is required by available methods. We then applied this protocol to breast cancer patient laser microdissected tissues in two proof-of-concept experiments, identifying differences in histone marks in heterogeneous regions selected by either morphological evaluation or MALDI MS imaging.

**Conclusions:**

These results demonstrate that analyzing histone PTMs from very small tissue areas and detecting differences from adjacent tumor regions is technically feasible. Our method opens the way for spatial epi-proteomics, namely the investigation of epigenetic features in the context of tissue and tumor heterogeneity, which will be instrumental for the identification of novel epigenetic biomarkers and aberrant epigenetic mechanisms.

**Supplementary Information:**

The online version contains supplementary material available at 10.1186/s13148-021-01120-7.

## Background

In the last decade, increasing evidence has highlighted the relevant contribution of epigenetic mechanisms in the development of a number of different diseases, including cancer. In particular, altered levels of histone post-translational modifications (PTMs) have been found in a multitude of diseased states. In cancer, specific histone PTM changes have been proposed as general hallmarks of cancer [1, 2], as prognostic biomarkers [3–6] and as useful tools for cancer detection and diagnosis [7, 8]. The widespread presence of histone PTM aberrations in tumors is in accordance with the observation that the enzymes responsible for the addition and removal of histone PTMs are among the most frequently mutated class of genes in cancer [9]. These discoveries fueled the investigation of epigenetic mechanisms in cancer, and the development of an increasing number of inhibitors of histone modifying enzymes, many of which have already been approved for human use or are undergoing clinical trials [10]. Despite the great promise of epigenetic therapy and the efforts devoted towards the development of epigenetic drugs, a number of challenges remain. Many epigenetic mechanisms underlying cancer still need to be elucidated in order to identify novel targets, and sensitive biomarkers are needed to better guide patient diagnosis and their treatment with the most appropriate epigenetic drugs. In this scenario, an accurate and quantitative evaluation of histone PTM levels in clinical samples is becoming increasingly needed, as it can not only provide biomarkers useful for patient stratification, but also suggest possible novel epigenetic mechanisms altered in cancer, which could be targeted for therapy.

Although the epigenetic analysis of clinical samples has been conducted so far mostly using antibody-based approaches, mass spectrometry (MS) is a much preferred tool, as it can provide a comprehensive, unbiased and quantitative profiling of histone PTMs [11]. We have recently developed a series of histone enrichment methods that allow the MS-based analysis of histone modifications in patient-derived samples, including formalin-fixed paraffin-embedded (FFPE) tissues, frozen tissues and primary cells [12–15]. These approaches involve separate processing protocols for histone H3 and H4, which are typically digested in-gel [12] and in-solution [15], respectively, to maximize the coverage of histone acetylations and methylations. Although these methods have been successfully applied to clinical samples, allowing the identification of different epigenetic patterns in tumor versus normal tissues [2], and in breast cancer subtypes [16], the substantial amount of starting material needed remains a major limiting factor. In a previous work, we verified the feasibility of applying our approaches to manually macrodissected or laser microdissected areas, scaling down the preparation to approximately 0.5 million cells [13]. Although this amount is much reduced compared with that commonly used for cell lines, it is still frequently much more than what can be obtained from clinical samples. In addition, an even higher amount is required to comprehensively dissect histone H4 modifications from clinical samples using an in-solution digestion [15]. The ability to profile small amounts of samples would be required in a number of clinical applications, including the analysis of early cancer lesions, micrometastases and tumor heterogeneity.

With the goal of reducing the amount of starting material needed for MS-based histone PTM analysis, in this study we tested and implemented different digestion strategies and streamlined the protein extraction procedure from low-amount clinical samples, generating a protocol that allows the analysis of the most common histone PTMs from tissue areas corresponding to 1000 cells. By applying this protocol to patient-derived tissues, we demonstrate that analyzing histone PTMs from very small tissue area is technically feasible, opening the way for the investigation of epigenetic features in the context of tissue and tumor heterogeneity.

## Methods

### Cell culture

The breast cancer cell line MDA-MB-468 was grown in in Dulbecco's modified Eagle's medium (DMEM) (Lonza) supplemented with 10% South American (SA) FBS (Lonza). Human glioblastoma cells were obtained as previously described [17]. For the experiment shown in Fig. 2, MDA-MB-468 were treated with DMSO or 15 nM Panobinostat (Sigma-Aldrich, in DMSO) for 4 h.

### Patient tissue specimens

The patient samples shown in Fig. 4 were obtained from the Biobank for Translational Medicine Unit (B4MED) of the European Institute of Oncology. Sample collection by the Biobank, in the presence of patient consent, was approved by the Ethical Committee of the European Institute of Oncology on June 6, 2011, and the samples can be used for research purposes, including future uses, without any further approval by the Ethical Committee [18]. Histone H3 PTMs from fresh frozen breast cancer and FFPE ovarian and head and neck cancers were already profiled in [2]. The FFPE samples shown in Fig. 5 were retrospectively obtained from the archive of the Istituto Nazionale Tumori of Milan, in compliance with the Internal Review Boards procedures. The levels of hormone receptors, Her-2 and Ki-67 were ascertained by immunohistochemistry and breast cancer subtypes were defined as described [19]. The samples were evaluated by a trained pathologist. Tumor-infiltrating lymphocytes (TILs) were completely surrounded by tumor cells, and long-distance lymphocytes (LDLs) were on the edge of the tumor, with at least partial exposure to stroma and/or normal cells.

### Laser microdissection (LMD)

For the experiments shown in Fig. 3, 4 µm-thick FFPE sections or 10 µm-thick snap-frozen OCT-embedded cryosections of mouse adult pancreas were mounted on polyethylene naphthalate membrane (PEN) slides (Leica No. 11600289) previously UV-photoactivated in a UV crosslinker for 30 min (BLX-254, Bio-Link). FFPE sections were de-paraffinized with two changes of xylene, while OCT-embedded sections were fixed in cold anhydrous ethanol for 3 min before proceeding to partial rehydration in graded alcohols up to 50%. Sections were then counterstained for 30 s with freshly prepared alcoholic-based buffered cresyl violet (0.8% cresyl violet in 60% EtOH and 4 mM Tris–HCl, pH8.0), washed twice in 75% EtOH and air-dried. Area of pancreatic tissues corresponding to ~ 20,000, 5000, 2500 and 1000 acinar cells was microdissected using a UV-based LMD7 LMD system (Leica Microsystems), collected into the caps of 0.5 ml PCR tubes and stored at 4 °C until further processing. Experimental procedures involving animals were performed in accordance with the Italian Laws (D.lgs. 26/2014), which enforces Dir. 2010/63/EU (‘‘Directive 2010/63/EU of the European Parliament and of the Council of 22 September 2010 on the protection of animals used for scientific purposes’’). All animal procedures were approved by the OPBA (Organismo per il Benessere e Protezione Animale) of the Cogentech animal facility at the IFOM-IEO Campus, Milan, and authorized by the Italian Ministry of Health.

For the experiment shown in Figs. 4 and 5, 10 µm-thick sections from fresh-frozen or FFPE breast cancer sample were mounted on PEN slides and stained with hematoxylin or cresyl violet. Areas corresponding to normal epithelial cells, infiltrating carcinoma, TILs or lymphocytes outside the tumor region (LDLs) were collected by LMD as described above.

### Histone enrichment

Histones were enriched from primary glioblastoma cells by resuspending 0.5–2 × 10^6^ cells in 1 ml of PBS buffer containing 0.1% Triton X-100 and protease inhibitors. Nuclei were isolated through a 10-min centrifugation at 2300×*g*, resuspended in 100 µl of the same buffer containing 0.1% SDS and incubated for few minutes at 37 °C in the presence of 250 U of benzonase to digest nucleic acids. Histones were purified from the MDA-MB-468 cell line through the same protocol, with the addition of an acidic extraction step [14]. OCT and FFPE whole sections (10 and 4 µm thick, respectively) were collected in 1.7-ml tubes and processed as described in [14]. The yield of histones deriving from the different purification protocols was estimated by SDS-PAGE gel by comparison with known amounts of recombinant histone H3.1, following protein detection with colloidal Coomassie staining (Expedeon).

Tissue pieces obtained by LMD were transferred at the bottom of the tubes through a 3-min centrifugation at maximum speed, and histones were enriched by adapting previously developed methods [14] to low sample amounts. FFPE microdissected tissue pieces were deparaffinized once in 200 µl hystolemon (Carlo Erba) and rehydrated in the same volume of solutions containing decreasing concentrations of ethanol (50% and 20% ethanol and water). The same rehydration steps were also performed for the OCT microdissected samples. Then, all the samples were resuspended in 35–40 µl 20 mM Tris pH 7.4 containing 2% SDS and homogenized by sonication in a Bioruptor device, through 10 cycles (30 s on/30 s off), at high potency. For FFPE samples, proteins were extracted and de-crosslinked at 95 °C for 45 min and 65 °C for 4 h.

### Histone digestion

Prior to digestion, the samples were mixed with a heavy-labeled histone super-stable isotope labeling by amino acids in cell culture (SILAC) mix, which was generated as previously described and used as an internal standard for quantification [19, 20]. When enough material was available, about 5 μg of histones per run per sample was mixed with an approximately equal amount of super-SILAC mix and separated on a 17% SDS-PAGE gel. The samples obtained from laser microdissected mouse pancreas were entirely loaded on a gel and mixed with 1 μg of super-SILAC mix. For in-gel digestions, a band corresponding to the histone octamer (H3, H4, H2A, H2B) was excised, chemically acylated with D_6_-acetic anhydride (D3-Ac protocol) or propionic anhydride (PRO, PRO-PRO and PRO-PIC protocols) and in-gel digested with trypsin (the combination of chemical acylation and trypsin digestion generates an “Arg-C-like” digestion). The digestion was performed as previously described [19], except that the extraction of the digested peptides from the gel was performed with acetonitrile 50% and 100%, without formic acid, which would impair the subsequent derivatization steps. In addition, for the PRO-PRO and PRO-PIC protocol, after elution the samples were concentrated to a volume below 3 µl, diluted to 9 µl with water and derivatized with phenyl isocyanate (PIC) or propionic anhydride. The derivatization with PIC was initially performed as described [21]. The samples were buffered to pH 8.5 by adding 1 μl of 1 M triethylammonium bicarbonate buffer, 3 μl of a freshly prepared 1% v/v PIC solution in acetonitrile was added (17 mM final concentration), and the mixture was incubated for 60 min at 37 °C. Finally, the samples were acidified by addition 8 μl of 1% trifluoroacetic acid (TFA). Then, because we observed that a relevant portion of peptides was not derivatized, we increased the incubation time to 1.5 h and increased the amount of PIC used (3 μl of a 5% v/v PIC solution in acetonitrile) (Additional file 1: Fig. S5). Derivatization with propionic anhydride was performed by adding 1 μl of a 1:100 dilution of propionic anhydride in ddH_2_O, vortexing and incubating for 2 min at room temperature. The reaction was stopped by adding 1 μl of 80 mM hydroxylamine for 20 min at room temperature and the samples were acidified with TFA, as described above [21]. Arg-C in-solution digestions were performed according to the manufacturer’s protocol, overnight at 37 °C. All samples were desalted on handmade StageTips, as previously described [19].

### LC–MS/MS analysis of histone PTMs

Peptide mixtures were separated by reversed-phase chromatography on an EASY-nLC 1200 high-performance liquid chromatography (HPLC) system through an EASY-Spray column (Thermo Fisher Scientific), 25 cm long (inner diameter 75 µm, PepMap C18, 2 µm particles), which was connected online to a Q Exactive HF or a Q Exactive Plus (Thermo Fisher Scientific) instrument through an EASY-Spray™ Ion Source (Thermo Fisher Scientific). Solvent A was 0.1% formic acid (FA) in ddH_2_O, and solvent B was 80% ACN plus 0.1% FA. Peptides were injected in an aqueous 1% TFA solution at a flow rate of 500 nl/min and were separated with a 50-min linear gradient of 0–35% solvent B for in-gel digested samples (gradient 1), or a 50-min linear gradient of 10–45% for PRO-PIC digested samples (gradient 2). A 50-min linear gradient of 0–30% was used for Arg-C digested samples. The Q Exactive instruments were operated in the data-dependent acquisition (DDA) mode to automatically switch between full scan MS and MS/MS acquisition. Survey full scan MS spectra (*m*/*z* 300–1350) were analyzed in the Orbitrap detector with a resolution of 60,000–70,000 at *m*/*z* 200. The 10–12 most intense peptide ions with charge states comprised between 2 and 4 were sequentially isolated to a target value for MS1 of 3 × 10^6^ and fragmented by HCD with a normalized collision energy setting of 28%. The maximum allowed ion accumulation times were 20 ms for full scans and 80 ms for MS/MS, and the target value for MS/MS was set to 1 × 10^5^. The dynamic exclusion time was set to 10 s, and the standard mass spectrometric conditions for all experiments were as follows: spray voltage of 1.8 kV, no sheath and auxiliary gas flow.

### Histone PTM data analysis

Acquired RAW data were analyzed using the integrated MaxQuant software v.1.5.2.8. The Uniprot HUMAN_histones 1502 database was used for histone peptide identification. Enzyme specificity was set to Arg-C. The estimated false discovery rate (FDR) of all peptide identifications was set at a maximum of 1%. The mass tolerance was set to 6 ppm for precursor and fragment ions. One missed cleavage was allowed, and the minimum peptide length was set to 4 amino acids. Variable modifications for in-solution Arg-C digestions were lysine monomethylation (+ 14.016), dimethylation (+ 28.031 Da), trimethylation (+ 42.046 Da) and acetylation (+ 42.010 Da). Variable modifications for the D3-Ac and PRO protocols include D_3_-acetylation/propionylation (+ 45.0294/+ 56.0262 Da), lysine monomethylation with D_3_-acetylation/propionylation (+ 59.0454/+ 70.0422), dimethylation, trimethylation and lysine acetylation. Variable modifications for the PRO-PIC and PRO-PRO protocols include lysine propionylation, monomethylation–propionylation, dimethylation, trimethylation and acetylation, and *N*-terminal PIC labeling (+ 119.0371 Da) or *N*-terminal propionylation (+ 56.0262 Da), respectively. To reduce the search time and the rate of false positives, which increase with increasing the number of variable modifications included in the database search [22], the raw data were analyzed through multiple parallel MaxQuant jobs [23], setting different combinations of variable modifications. Peptides with Andromeda score less than 60 and localization probability score less than 0.75 were removed. Identifications, retention times and elution patterns of isobaric peptides were used to guide the manual quantification of each modified peptide using QualBrowser version 2.0.7 (Thermo Fisher Scientific). Site assignment was evaluated using QualBrowser and MaxQuant Viewer from MS2 spectra. Extracted ion chromatograms (XICs) were constructed for each doubly charged precursor, based on its *m/z* value, using a mass tolerance of 20 ppm and a mass precision up to four decimals. Data from the PRO-PIC experiments were searched using EpiProfile 2.0, which performs peak extraction and XIC quantification in an automated manner [24]. For each histone modified peptide, the % relative abundance (%RA) was estimated by dividing the area under the curve (AUC) of each modified peptide for the sum of the areas corresponding to all the observed forms of that peptide and multiplying by 100 [25]. For SILAC experiments, Arg10 was selected as heavy label (multiplicity = 2) in MaxQuant or EpiProfile 2.0. The heavy form of each modified peptide was quantified from its XIC and the relative abundance calculated. The AUC values for all the samples analyzed are reported in Additional file 2: Dataset S1. The mass spectrometry proteomics data have been deposited to the ProteomeXchange Consortium [26] via the PRIDE partner repository with the dataset identifier PXD024799 and PXD024745.

### MALDI-mass spectrometry imaging (MSI)

Tissue sections of 12 μm thickness were thaw-mounted onto poly-l-lysine-coated (Sigma-Aldrich, Saint Louis, MO, USA) indium-tin oxide (ITO) conductive slides (Bruker Daltonics, Billerica, MA, USA) and stored at − 80 °C until use. ITO slides were thawed under vacuum for 15 min, and 8 layers (2 layers at 5 µl/min followed by 6 layers at 10 µl/min) of 2,5-dihydroxybenzoic acid (DHB (Sigma-Aldrich, Saint Louis, MO, USA) 30 mg/ml in MeOH:H_2_O 70:30, 0.2% TFA) were applied using a SunCollect (SunChrom, Friedrichsdorf, Germany) automated matrix spraying system (4 bar, 50 mm *z* axis height). The tissue sections were then dried under vacuum for 15 min prior to MALDI MSI data acquisition. MALDI MSI was performed with an EP-MALDI source [27] (Spectroglyph, LLC., Kennewick, WA, USA) equipped with a 349-nm laser (Spectra-Physics, Santa Clara, CA, USA), coupled with an Orbitrap QExactive Plus (Thermo Fisher Scientific). The laser was operated at 1.65 A and 500 Hz, the ion source pressure was 7.2 Torr, and the MSI pixel size was 35 × 35 µm. Mass spectra were acquired in the range 150–2000 m/z at 70,000 resolving power. The position file was aligned to the raw file using Image Insight (v. 0.1.0.11550, Spectroglyph, LLC).

### Image pre-processing and data analysis

The raw spectra of the MSI dataset were first converted into mzXML using RawConverter [28]. The MSI datacube was produced from the mzXML files and position files using the script ORBIIMAGEmzXML2Tricks (v. 0.10, G. Eijkel). MSI datacubes were imported into MATLAB R2019b (MathWorks, Natick, MA, USA) for image preprocessing and data analysis. The tissue area was manually selected and features that negatively correlated to the tissue mask were discarded. Mass spectral features were de-isotoped using an in-house coded script, and the intensities of non-tissue pixels were set to zero. The resulting datacube was TIC normalized, and bright spots with an intensity over the 99.9th percentile were removed. The MALDI image was weakly denoised using an edge-preserving [29] total variation minimizing Chambolle algorithm (*λ* = 0.1, 1 iteration) applied at the m/z level. Unsupervised k-means cluster analysis was performed on the tissue pixels only (10 clusters, cosine distance, three replicates).

### Statistical analysis

Data display was carried out using Perseus [30] and GraphPad Prism 8.2.1 (Graphpad). Statistical testing was performed using GraphPad Prism. Changes in individual modified peptides between multiple groups were evaluated by one-way ANOVA (data shown in Fig. 4) or by repeated-measures ANOVA (data shown in Fig. 5), followed by Tukey’s multiple comparison test. Normalized *L*/*H* ratios, defined as *L*/*H* ratios of relative abundances normalized over the average value across the samples, were visualized and clustered with correlation distance and average linkage as parameters.

## Results

### Comparison of in-gel derivatization procedures

**Fig. 1:**
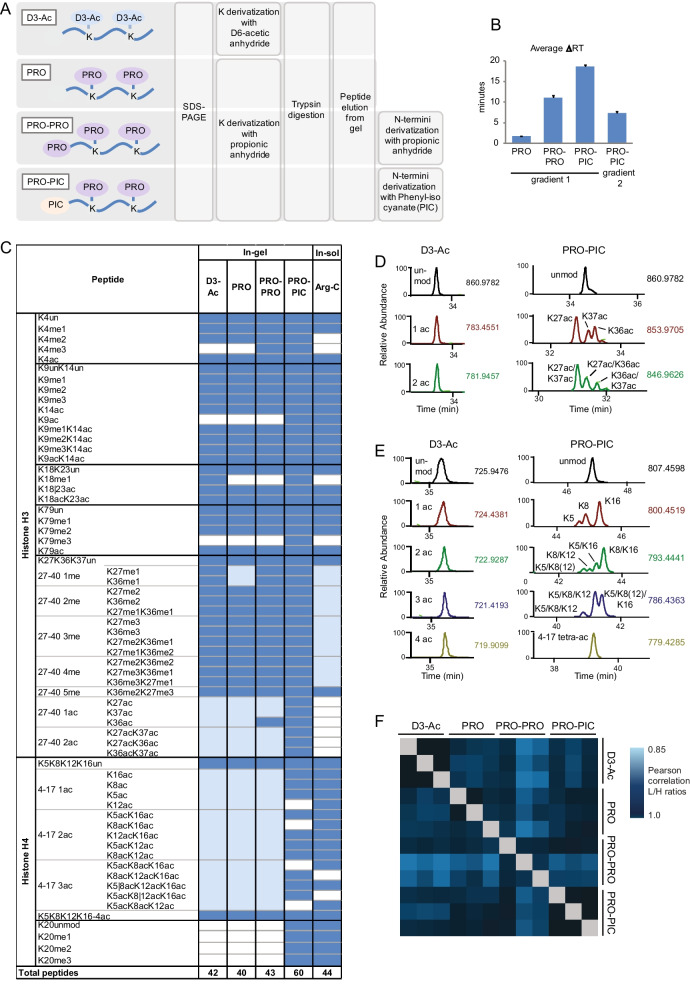
Comparison of in-gel digestion methods for MS-based histone PTM analysis. **a** Schematic representation of the protocols used to in-gel digest histones. **b** Average chromatographic retention time drifts for peptides obtained from the digestion of MDA-MB-468 cells with the four methods, as compared with the D3-Ac method. Two different gradients were tested for the PRO-PIC method (see Additional file 1: Fig. S4). **c** List of peptides identified and quantified from MDA-MB-468 cells using the four different in-gel or the Arg-C in-solution digestion protocols, which were performed in technical triplicates. The lighter blue color indicates isobaric peptides that could not be quantified individually. **d** and **e** Elution profiles of the differentially acetylated forms of the H3 27–40 peptides (**d**) and H4 peptide 4–17 (**e**) for samples digested in-gel using the D3-Ac or the PRO-PIC protocol. **f** Correlation matrix based on Pearson correlation coefficients of *L*/*H* ratios (light channel: MDA-MB-468 cells; heavy channel: spike-in standard) for histone PTMs quantified from samples processed in technical triplicates through the four in-gel digestion protocols

In order to optimize the histone digestion procedure for low-abundance clinical samples, we compared different in-gel digestion protocols. We chose to focus on an in-gel, rather than an in-solution, protocol because SDS-PAGE serves both to eliminate the detergents and other MS contaminants (e.g., the optimal cutting temperature (OCT) compound) present in the histones enriched from clinical samples, and to separate the histones from the other proteins present in the extracts [14]. Histones are usually digested in-gel using “Arg-C-like” strategies, which involve chemical acylation of lysines followed by trypsin digestion [31]. Acylated lysines are not recognized by trypsin, which—as a consequence—cuts only at the C terminus of arginines and generates peptides of suitable length for MS analysis. As an added advantage, because acylation occurs only on unmodified and mono-methylated lysines, it causes shifts in the retention times of isobaric peptides, which can be exploited for the quantifications of complex peptides carrying multiple modifications, such as the H3 27–40 peptide [16, 30] (Additional file 1: Fig. S1). Our reference protocol for in-gel digestion of histones employs deuterated acetic anhydride as acylating agent [32] (Fig. 1a* D3 protocol*). As an alternative to the deuterated acetic anhydride used in D3 protocol, we tested another widely used acylating agent, propionic anhydride (Fig. 1a, *PRO protocol*), also in combination with a second round of derivatization with propionic anhydride (Fig. 1a, *PRO-PRO protocol*) or phenyl isocyanate (PIC) (Fig. 1a, *PRO-PIC protocol*). The second derivatization step is performed after trypsin digestion and modifies the peptide *N* termini, increasing peptide hydrophobicity and improving peptide reversed-phase chromatographic retention. In particular, derivatization with PIC has proved to improve the detectability and the chromatographic retention of short and hydrophilic peptides (for instance the histone H3 3–8 peptide). In addition, this strategy has been shown to increase the detection of low-abundance acetylations, such as H3K27ac and H3K36ac [21]. Although all these strategies have already been described for in-solution digestions [21, 33], in this study we adapted them to an in-gel digestion, as described in the Material and Methods section.

We processed 5 µg of nuclear extracts obtained from glioblastoma patient-derived neurospheres and MDA-MB-436 cells using the four methods (Additional file 1: Fig. S2) and compared the modified peptides in terms of retention times and intensities. As expected, all the derivatization methods tested caused an increase in peptide retention times compared with the D3 protocol, with the PRO-PIC method showing the highest increase (Fig. 1b, Additional file 1: Fig. S3A, B), when using the same chromatographic gradient (gradient 1). Because this gradient does not allow the proper separation of the different forms of the H3 peptide 73–83 derivatized with PRO-PIC, for this protocol we used a slightly different gradient, which started from a higher percentage of organic solvent (gradient 2) (Fig. 1b, Additional file 1: Fig. S4). In addition, because we observed that a relevant portion of peptides did not carry PIC when applying the published derivatization procedure [21] to peptides eluted from the gel, we optimized the amount of PIC and the incubation time to obtain a more extensive derivatization (see the Material and Methods section and Additional file 1: Fig. S5). The PRO-PIC method showed the highest intensities for most peptides, with a remarkable increase for peptides that are commonly challenging to detect (the differentially modified forms of the H3 27–40 peptide and H3K4me2/me3, Additional file 1: Fig. S3C). Figure 1c shows the list of the most common histone H3 and H4 modified peptides identified and quantified with the four in-gel digestion methods. Because the Arg-C in-solution digestion is the most suitable approach to study histone H4 PTMs [32], it was also included as a reference. The protocols involving a single derivatization step perform similarly, while the second derivatization step appears to provide an advantage in terms of number of quantifiable peptides. With 60 distinct differentially modified peptides quantified, the PRO-PIC method performs by far the best among the four methods tested (Additional file 1: Figs. S6–S12). More specifically, thanks to the retention time shift among isobaric peptides, it allows quantifying different acetylations that cannot be distinguished with the other methods, such as H3K9ac (Additional file 1: Fig. S7), the distinct acetylated forms of the H3 27–40 peptide (H3K27ac, H3K36ac and H3K37ac, Fig. 1d and Additional file 1: Fig. S9) and 12 differentially acetylated forms of the histone H4 N-terminal tail (Fig. 1e and Additional file 1: Fig. S11). The PRO-PIC method also allows quantifying the modifications on the H4K20 residue (Additional file 1: Fig. S12), which cannot be detected using the other in-gel digestion methods, and are usually analyzed through an Arg-C in-solution digestion. Importantly, the number of modified peptides quantified by using the PRO-PIC approach alone is higher than the number of peptides quantified by combining any other in-gel digestion methods and the Arg-C in solution digestion, thus providing a more efficient strategy, in terms of both processing time and amount of material needed. From the quantitative point of view, the results obtained with the four in-gel digestion methods were remarkably similar (Fig. 1f, Additional file 1: Fig. S13), when coupled with the use of a spike-in heavy labeled super-SILAC standard [20] (results are expressed as ratios between sample and standard).

### Quantification of histone PTMs in low abundance samples

**Fig. 2:**
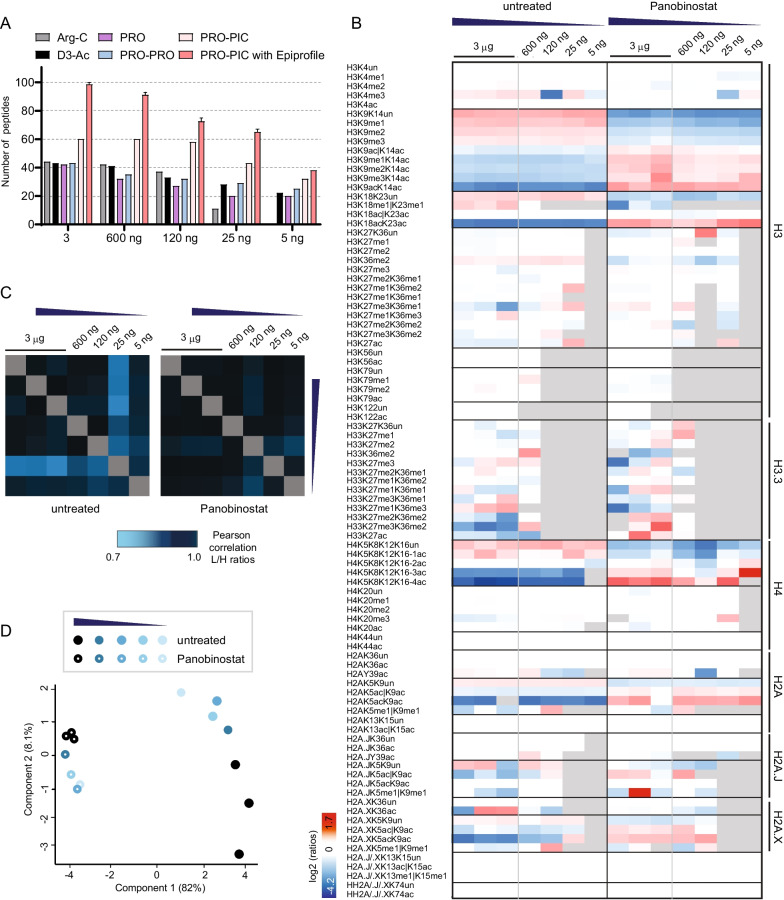
Performance of digestion methods for the analysis of histone PTMs from  low-abundance samples. **a** Number of differentially modified peptides quantifiable from samples digested using the four in-gel or the Arg-C in-solution digestion protocols, at decreasing peptide amounts. After digestion and prior to MS analysis, the peptides were subjected to 1:5 serial dilutions, from an approximate starting amount of 3 µg to 5 ng (1:625 dilution). The PRO-PIC samples were quantified either manually (light red), or with the EpiProfile 2.0 software, followed by manual validation (dark red). **b** Heatmap display of the log_2_ of ratios obtained for the indicated histone PTMs in MDA-MB-468 cells left untreated or treated with the HDAC inhibitor Panobinostat at 15 nM. The peptides were subjected to 1:5 serial dilutions, as described in (**a**). Grey: not quantified. **c** and **d** Correlation matrix based on Pearson correlation coefficients of *L*/*H* ratios (**c**) and principal component analysis (PCA,  **d**) for histone PTMs quantified from the MDA-MB-468 samples shown in (**b**)

In order to test the performance of the different digestion methods in the presence of small sample amounts, we performed serial 1:5 dilutions (from 3 µg, to approximately 5 ng) of the peptides obtained from the digestion of MDA-MB-436 cells (Fig. 2a, Additional file 1: Fig. S14). The Arg-C digestion was the most affected by low amounts of material, with only 11 peptides identified from approximately 25 ng of starting material. Instead, many peptides digested in-gel were detectable from amounts of injected material as low as 5 ng. One exception is represented by the H3 27–40 peptide, which was the first to disappear in all the tested conditions. This behavior is likely due to the fact that this peptide exists in many different modified forms, and is typically more challenging to analyze. Once again, the PRO-PIC method displayed the best performance: 43 peptides could be quantified from 25 ng of injected material and 32 from 5 ng. These numbers could be further increased by searching additional specific sites/modifications of interest. For instance, when using the EpiProfile 2.0 software, a computational platform for the automatic extraction and quantification of peaks corresponding to modified histone peptides and which supports derivatization with the PRO-PIC reagents [24], additional and less common modified peptides from histones H3, H3.3, H4 (e.g., modifications on H3K56, H3K122 and H4K44) and different variants of H2A could be quantified (Fig. 2a). While some of the new peptides/modifications were detectable only in the presence of high starting amounts of material, others could be detected from as low as 5 ng of injected peptides. In total, 85 peptides were quantified using the EpiProfile 2.0 software from 3 µg of injected material, which can be further increased to approximately 100 peptides by manually quantifying isobaric peptides, such as H3K27/36/37ac and the *N*-terminal histone H4 acetylated peptides (Fig. 2a, Additional file 1: Fig. S14). This number was reduced with decreasing amount of analyzed material, but, remarkably, 31 (38 including manual quantification) peptides could still be quantified from 5 ng of material (Fig. 2a). When using the EpiProfile software to quantify histone PTMs from MDA-MD-436 cells treated either with DMSO or the histone deacetylase (HDAC) inhibitor Panobinostat, we observed an extremely high (> 0.9) peptide ratio correlation across biological triplicates and dilutions (Fig. 2c), suggesting the quantification of histone PTMs, when present, is very accurate even with low sample amounts. The highest variability was observed in peptide 27–40 of histone H3 variant H3.3. Such variability can be explained considering the low abundance of histone H3.3, which is reflected in an intensity 10–15 times lower of the H3.3 27–40 peptide compared with the same peptide of canonical histone H3.1/2 (Additional file 2: Dataset S1). Of note, the peptides shown in Fig. 2b might be slightly different from those shown in Additional file 1: Fig. S14, because the peptides should be quantified both in the light and heavy channels. Samples not treated and treated with Panobinostat were clearly separated in a principal component analysis (PCA), even considering the lowest amount of material (Fig. 2d). As expected, treatment with the HDAC inhibitor caused a general increase in acetylated peptides and a decrease in non-acetylated peptides, although several acetylations were not affected (e. g. some of the acetylations on histone H2A variants, Fig. 2b). Interestingly, some changes could also be observed at specific methylation sites, such as the H3K36me2 mark (Fig. 2b), indicating a crosstalk between this modification and histone acetylation, and highlighting how MS-based profiling can provide a bird's-eye view on histone PTMs levels, revealing unexpected changes.

### Quantification of histone PTMs from laser microdissected samples

**Fig. 3:**
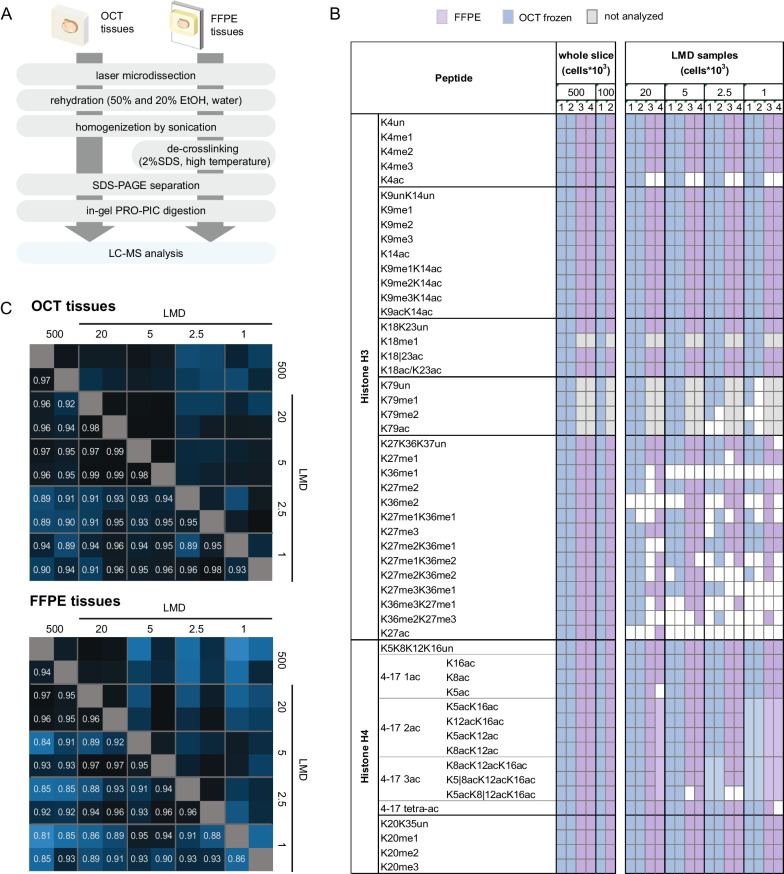
Quantification of histone PTMs from mouse pancreas laser microdissected samples. **a** Schematic representation of the protocols used to isolate histones from laser microdissected OCT or FFPE samples. **b** List of differentially modified histone peptides identified and quantified from manually macrodissected areas corresponding to approximately 500,000 or 100,000 cells, or laser microdissected areas (20,000, 5000, 2500 and 1000 cells) processed using the in-gel PRO-PIC digestion protocol. Four samples—comprising both FFPE and OCT tissues-were analyzed for each condition, with the exception of the 100,000 condition, which was acquired in duplicate. White spaces indicate that the peptide was not quantified. **c** Correlation matrix, based on Pearson's correlation coefficients (shown in the matrix) of log2 (*L*/*H* ratios) values for differentially modified histone peptides in the whole slice and LMD samples shown in (**b**)

To verify the performance of the PRO-PIC in-gel digestion method in a setting that better mimics the processing of low amounts of cells/tissues, which typically leads to relevant loss of material throughout the procedure, we processed different amounts of mouse pancreatic tissue. Areas containing approximately 500,000 and 100,000 cells were manually macrodissected, while tissue pieces containing 20,000, 5000, 2500 and 1000 were obtained by laser microdissection (LMD). We used tissue stored as formalin-fixed paraffin-embedded (FFPE) or OCT-embedded frozen samples, to account for possible differences due to storage conditions. FFPE is the most common form of preservation of patient specimens, and FFPE archives represent a precious source of retrospective clinical samples. Although formalin fixation can cause the appearance of artefactual modifications, we have shown that most of the best-characterized histone modifications can be accurately quantified from FFPE samples, with few exceptions [19]. OCT embedding is another, less common, storage method used in tissue biobanks. Frozen tissues are usually preferable for MS analyses compared with FFPE tissues, but the OCT compound must be carefully removed, as it is a strong MS contaminant. Tissue extracts were obtained using previously published protocols [14], which were implemented and simplified to minimize sample loss (Fig. 3a). Although not all the forms of the histone H3 peptide 27–40 can be quantified from 20,000 cells or less, most of the other modified peptides were detected from all the cell amounts tested (Fig. 3b). The correlation of peptide ratios was very high for all the conditions, although slightly lower for FFPE compared with OCT-frozen tissue (Fig. 3c), indicating that the workflow involving the PRO-PIC digestion has the potential to be applied to very small tissue areas.

### Quantification of histone PTMs from patient breast cancer laser microdissected tissue

**Fig. 4:**
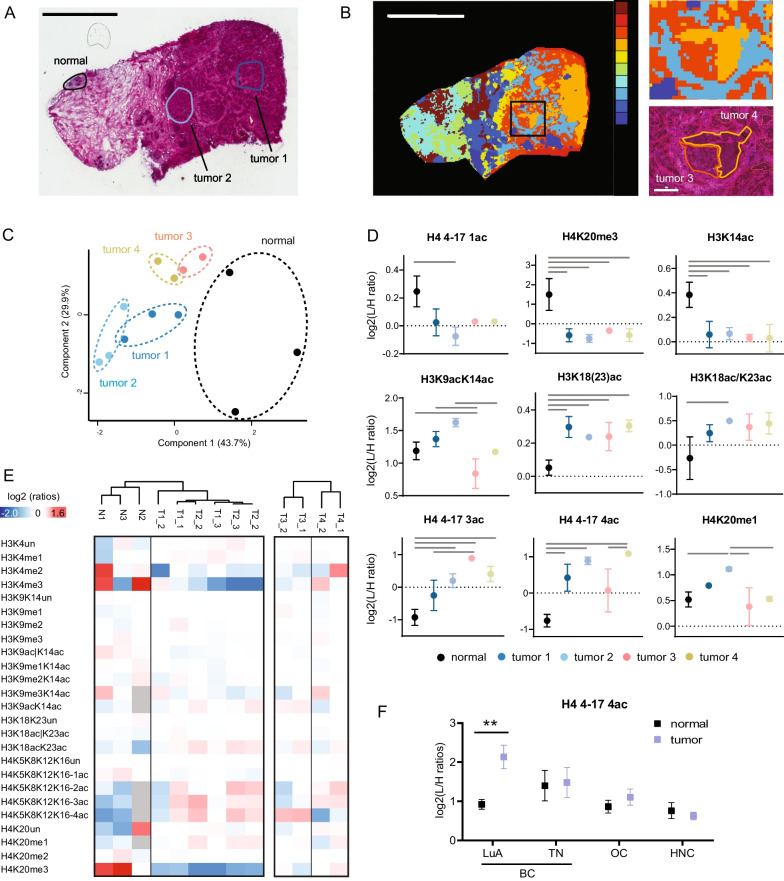
Histone PTM quantification from breast normal and tumor samples. **a** H&E staining of a Luminal A-like breast cancer section. Normal epithelial cells and tumor cells (areas containing 1800–4500 cells from four consecutive sections) in the indicated areas were collected by LMD and analyzed by MS. Scale bar: 2 mm. Of note, histone H1 variants were analyzed from the same tissue areas in a previous publication [36]. **b** Unsupervised tissue segmentation (k-means clustering, 10 clusters) based on the MALDI MSI data. Scale bar 2 mm. In the magnification, the detail of the clusters used to define tumor 3 and tumor 4 regions, with the corresponding histological image. Scale bar 330 µm. **c** PCA analysis based on histone PTM data obtained from the normal and tumor areas highlighted in** a**,** b**. **d** Graphs showing significant differences among the tissue areas collected by LMD. Error bars represent the standard error of the mean (SEM) from 2 to 3 LMD areas. Samples were compared by one-way ANOVA, followed by multiple comparison test. The lines indicate a *p* value < 0.05, with the exception of the comparison of the H4 4–17 4ac peptide between tumor 3 and 4, where *p* = 0.07. **e** Heatmap display of the log2 of L/H ratios obtained with the super-SILAC strategy (light channel: laser microdissected sample, heavy channel: spike-in standard) obtained for the indicated histone PTMs in normal and tumor samples. The heatmap on the left shows the results for the tissue areas selected by the pathologist, the one on the right the areas selected by MALDI-MSI. The samples were normalized over the average value across all the samples shown in each heatmap. The grey color indicates peptides that were not quantified. **f** Graphs showing the levels of the H4 4–17 4ac peptide in normal and tumor samples for Luminal A-Like breast cancer (BC) samples (*n* = 5, matched), triple-negative BC samples (*n* = 3, matched), ovarian cancer (OC) and head and neck cancer (HNC). Error bars represent the standard error of the mean (SEM). Samples were compared by multiple t test (**FDR < 0.01)

Next, we applied the isolation protocol from frozen tissue combined with the PRO-PIC in-gel digestion method to patient breast cancer samples that were subjected to LMD, in two proof-of-concept experiments. In Fig. 4, we compared normal and infiltrating carcinoma areas containing approximately 700–4500 cells (corresponding to an area of 0.11–0.68 mm^2^), which were laser microdissected from a breast cancer patient specimen belonging to the Luminal A-like molecular subtype. Specific areas were microdissected from the normal and tumor regions of each sample. Two of the tumor areas were selected based on different morphological features, as judged by the evaluation of a trained pathologist (tumor 1 and 2, Fig. 4a). Two additional tumor areas (tumor 3 and 4, Fig. 4b) were selected based on the lipid molecular profiles obtained by matrix-assisted laser desorption/ionization mass spectrometry imaging (MALDI-MSI), which provides information on the spatial distribution of hundreds of analytes on the tissue section, and defines molecular differences which may be missed by a morphological evaluation [34]. When superimposed on the H&E-stained section, the tumor regions defined by MALDI-MSI appear to correspond to tumor areas with different amounts of stroma (Fig. 4b). The PCA analysis based on histone PTM profiles separated the normal and tumor microdissected tissue samples and generated defined clusters comprising the replicate areas analyzed for tumors 1–4 (Fig. 4c). As expected, in tumors we detected a significant decrease of H3K14ac, of the mono acetylated form of histone H4 and of H4K20me3, the loss of which has been reported as a general hallmark of cancer [1, 2] (Fig. 4d, e). In addition, we detected a decrease in acetylations on the H3 18–26 peptide in all the tumor regions, while the levels of acetylations on H3K9/K14 and on the histone H4 tail were more heterogeneous. In particular, although being generally increased compared with the normal tissue, the tetra-acetylated form of histone H4 4–17 peptide showed different levels across tumor samples, and even between tumor samples 3 and 4, despite the close proximity of these two regions (Fig. 4d, e). The increase in H4 4–17 4ac-peptide in breast cancer was confirmed in a dataset composed of 5 normal and tumor matched fresh-frozen Luminal A-like breast cancer samples, where LMD was not performed (Fig. 4f). Interestingly, no change in this peptide was observed in breast cancer belonging to the triple-negative subtype, nor in ovarian or head and neck tumors, compared with their normal counterparts. These results suggest that increase in hyper-acetylation of the histone H4 tail not only appears to be tumor subtype specific, but also it can distinguish different areas within the same tumor.

**Fig. 5:**
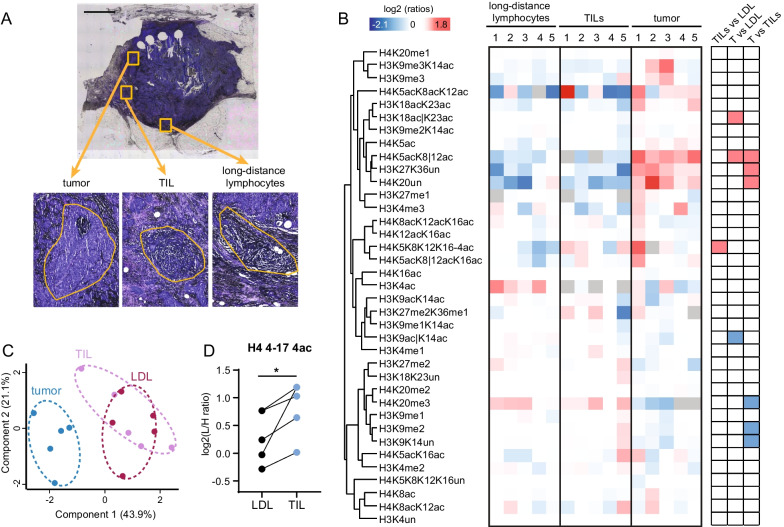
Histone PTM quantification from breast tumor samples and lymphocytes. **a** Crystal violet staining of a representative breast cancer section. Tumor cells (T), tumor-infiltrating lymphocytes (TILs) and lymphocytes outside of the tumor region [long-distance lymphocytes (LDLs)] were collected by LMD in five patients and analyzed by MS. Scale bar: 2 mm. **b** Heatmap display of the log2 of ratios obtained for the indicated histone PTMs in tumor cells, TILs and LDL in the samples described in (**a**). The *L*/*H* ratios of relative abundances obtained with the super-SILAC strategy (light channel: laser microdissected sample, heavy channel: spike-in standard) normalized over the average value across all samples are shown. The grey color indicates peptides that were not quantified. Right panel: modified peptides were compared by repeated measures ANOVA, followed by Tukey’s multiple comparison test. The red color indicates an increase, the blue color a decrease (*p* < 0.05). **c** PCA analysis based on histone PTM data obtained from tissue areas highlighted in (**a**). **d** Plot showing the levels of the tetra-acetylated form the H4 4–47 peptide in TILs and LDLs. Significance was assessed as in (**b**)

In a separate experiment, we analyzed tissue heterogeneity in sections obtained from triple-negative breast cancer patients by selecting areas containing tumor cells, tumor-infiltrating lymphocytes (TILs) and lymphocytes outside of the tumor region (defined here as long-distance lymphocytes, LDLs) (Fig. 5a). The analysis of 5 patients highlighted several differences between tumor and lymphocytes (Fig. 5b), which were separated by PCA analysis (Fig. 5c). In tumor cells, we found decreased levels of H4K20me3 and a marked increase in the unmodified forms of the H3 27–40 and H4 20–24 peptides. These differences were also identified when comparing tumor and normal cells ([1, 2] and Fig. 4) and may be partially explained by the higher proliferation rates of the tumors [2]. In addition, tumor cells showed a marked increase in H4K5acK8|12ac compared with both TILs and LDLs. Interestingly, despite their different microenvironment, TILs and LDLs were instead much more similar to each other, with only one significant change, namely an increase in the tetra-acetylated form of the histone H4 tail in TILs (Fig. 5d).

Taken together, these results demonstrate that analyzing histone PTMs from very small tissue areas is technically feasible and that our method enables, for the first time, the quantitative analysis of tens of histone PTMs from microdissected samples/specific cells within the same patient tissue sample.

## Discussion

In recent years, there has been an increasing interest in the application of molecular approaches and—OMICS technologies in clinical applications. Dealing with patient-derived tissues, however, often poses the problem of limited sample amounts. This for example occurs when analyzing very early cancer lesions, tissue biopsies, micrometastases or tumors that shrunk after a successful therapy. Additionally, the investigation of specific cell populations (e.g., immune cells vs tumor cells) within a tissue, or of heterogeneous tumor areas requires the ability to handle very small sample amounts. Molecular biologists have overcome this problem with the development of methods to amplify nucleic acids, such as polymerase chain reaction. However, similar amplification methods do not exist for proteins. Therefore, extracting, separating and quantifying the proteins present in extremely small samples present significant challenges, mostly related with the manual manipulation of small volumes/tissue areas. A few methods have been proposed for the proteome profiling of microscopic tissue areas [35], but this subject has never been addressed for histone PTMs.

Our results from 2017 proved the possibility to perform a MS-based analysis of histone PTMs from laser microdissected samples [13], but the starting amount required (450,000 cells) was still limiting. In this study, we sought to further optimize the workflow for sample preparation of histones for PTM analysis, by streamlining the protein extraction protocol and optimizing the digestion step, with the goal of reducing the amount of starting material needed. Among the digestion methods tested, the in-gel PRO-PIC protocol works the best under many different aspects. First, it allows the quantitation of the higher number of modified peptides, most of which can be analyzed in an automated manner through the EpiProfile software [24]. It must be underlined that our analysis is not exhaustive, as we focused only on lysine acetylations and methylations, and could be expanded by performing searches tailored to the specific need/interest of the researcher. Second, the PRO-PIC in-gel digestion method allows saving precious tissue by avoiding the need for a separate in-gel “Arg-C-like” digestion, which is typically used to study histone H3 PTMs from 4–5 µg of histone, and an Arg-C in-solution digestion, which provides a better coverage of histone H4 PTMs [32]. In the case of clinical samples, this in-solution digestion must be preceded by protein precipitation and a Stage-Tip purification to remove detergents and MS contaminants, which requires at least 10 µg of histones [15]. Finally, the PRO-PIC protocol showed the best performance with low sample amounts, dramatically reducing the quantity of material needed for the analysis of histone PTMs. These results can be exploited not only for laser microdissected tissues and clinical samples available in small quantities, but also for other research samples available in limited amounts, such as flow-cytometry sorted cells or patient-derived primary cells. It should be noted that the detectability from low sample amounts changes dramatically for different peptides. For instance, the modified forms of the H3 27–40 peptides can be comprehensively profiled from a minimum of 100,000 macrodissected cells, while most forms of the H3 9–17 and 3–8 peptides are detectable from a remarkably low number of microdissected cells (1000 cells). Keeping in mind that the detectability of specific modifications depends on the abundance of that modification in a specific sample, our results provide a benchmark for the minimum amount of material/cells needed to analyze specific peptides or modifications. It will be interesting in the future to combine the PRO-PIC digestion with targeted MS methods and separation sciences (e.g., through µPAC columns for single cell analysis) to further increase detection sensitivity, and thus be able to assay even the most challenging peptides (e.g., H3 27–40) from ever smaller regions of tissue.

The ability to accurately profile a good proportion of the most common histone PTMs from as low as 1000 cells addresses an unmet need in the epigenetic field, namely the possibility to dissect tissue heterogeneity. Our method allows the analysis of specific cell populations or morphological features of limited size and opens the way for the investigation of histone PTMs in the context of intra-tumor heterogeneity. Tumors comprise a mixture of cell populations with distinct phenotypic and molecular features, which may respond differently to drug treatment, affecting patient outcome. The ability to profile histone PTM levels in tumor subpopulations, which is made possible for the first time by our method, represents an important step towards understanding the contributions of epigenetics in tumor resistance and improving response to therapy in heterogeneous tumors. We recently developed another MS-based method enabling the quantitation of histone H1 variants from low-abundance samples and laser microdissected tissue areas containing 1000 cells [36]. This approach, which measures histone protein levels rather than histone PTMs, employs a label quantitation strategy, differently from the spike-in method used in this study. The two pieces of information regarding histone PTMs and variants could be integrated to obtain a more complete picture of the epigenetic status of a clinical sample. Importantly, the two methods are compatible and can be performed from the same sample loaded on the gel, as they involve the digestion of bands corresponding to different molecular weight ranges. For example, normal and tumor 1 and 2 areas from the breast cancer sample shown in Fig. 4 were also subjected to histone H1 variant profiling, which highlighted significant differences [36]. In this study, we included two proof-of-concept experiments, where tissue heterogeneity was investigated at different levels: tumor versus adjacent normal tissues, tumor heterogeneity, and the presence of immune cells within the tissue section. All these analyses highlighted several differences in histone marks, which were more marked when comparing tumor and non-tumor cells, but also present among tumor regions or lymphocytes within or outside the tumor area.

MALDI-MSI is able to simultaneously record the distributions of a large number of analytes within patient tissues and has been widely investigated for cancer diagnosis, prognosis and response prediction [37]. Here we exploited the ability of MALDI-MSI to detect differences in molecular features that may not be evident by histopathological evaluation, specifically intratumor heterogeneity [38]. MSI has been used to uncover intratumor heterogeneity at the protein [38] and lipid levels [39]. Here, we used MALDI-MSI of lipids to demarcate specific tumor subpopulations, which were then isolated for epi-proteomics characterization to investigate if this heterogeneity also correlated with histone modifications. MALDI-MSI has also been used to directly investigate histones in tumor tissues; for instance, an ultrahigh mass resolution MALDI-MSI investigation revealed increased levels of acetylated histones H2A and H2B in glioblastoma with respect to the surrounding tissue [40]. However, even at the very high mass resolution afforded by the use of a 15 T Fourier transform ion cyclotron resonance mass spectrometer, it remains challenging to assign most of the mass spectral peaks to unique histone proteoforms, because of the preponderance of isobaric ions (identical nominal mass) and structural isomers (same number of modifications but different modification sites). The combination of MALDI-MSI, LMD and LC/MS analysis of histone PTMs used here enables a much higher depth of coverage of histone marks, leading to unequivocal assignment.

The epigenetic marks identified through our approach could be useful as biomarker or serve as the basis for further investigations of epigenetic mechanisms, possibly in combination with other—OMICS approaches, such as ChIP Seq, RNA-seq and proteomics analyses. One example is represented by the increase in H4 4–17 tetra-acetylated peptide, which was observed in Luminal A-like tumors compared with normal tissues—with differences among heterogeneous tumor regions—and in TILs compared with LDLs. High levels of TILs are associated with improved survival and better prognosis in many cancer types, including triple-negative breast cancers [41]. Increased levels of acetylation may represent an additional marker of activated immune cells within the tumor. In addition, it would be interesting to study the effects of histone H4 hyper-acetylation, which is typically associated with active transcription, on the expression of specific genes/pathways, and investigate whether they are involved in TIL’s anticancer activities or could be exploited to improve cancer treatment by immunotherapy.

## Conclusions

In this study, we tested and implemented different digestion strategies and optimized the protein extraction protocol from low-amount clinical samples, generating a protocol that allows the analysis of up to 38 differentially acetylated/methylated histone peptides from tissue areas corresponding to 1000 cells. This represents a remarkable improvement compared with available methods, as this amount is 500 times lower than what was required by our previously reported protocol. By applying this method to patient-derived tissues, we demonstrate that analyzing histone PTMs from very small tissue area not only is technically feasible, but also allows quantifying epigenetic differences among adjacent tissue areas. Thus, our method enables for the first time the investigation of epigenetic features in the context of tissue and tumor heterogeneity, addressing an unmet need in the epigenetic field and providing a tool that will facilitate the identification of epigenetic biomarkers and aberrant epigenetic mechanisms.

### Supplementary Information

**Additional file 1**. Supplementary tables and figures.

**Additional file 2. Dataset S1**. Histone PTM quantification data.

## Data Availability

The mass spectrometry proteomics data have been deposited to the ProteomeXchange Consortium [26] via the PRIDE partner repository with the dataset identifier PXD024799 and PXD024745.

## Abbreviations

FFPE: Formalin-fixed paraffin-embedded
OCT: Optimal cutting temperature
HDAC: Histone deacetylase
HPLC: High-performance liquid chromatography
LDLs: Long-distance lymphocytes
LMD: Laser microdissection
MALDI-MSI: Matrix-assisted laser desorption ionization-mass spectrometry imaging
PIC: Phenyl isocyanate
PCA: Principal component analysis
PTMs: Post-translational modifications
SILAC: Stable isotope labeling by amino acids in cell culture
TILs: Tumor-infiltrating lymphocytes

## References

[1] Fraga MF, Ballestar E, Villar-Garea A, Boix-Chornet M, Espada J, Schotta G (2005). Loss of acetylation at Lys16 and trimethylation at Lys20 of histone H4 is a common hallmark of human cancer. Nat Genet.

[2] Noberini R, Restellini C, Savoia EO, Raimondi F, Ghiani L, Jodice MG (2019). Profiling of epigenetic features in clinical samples reveals novel widespread changes in cancer. Cancers (Basel).

[3] Elsheikh SE, Green AR, Rakha EA, Powe DG, Ahmed RA, Collins HM (2009). Global histone modifications in breast cancer correlate with tumor phenotypes, prognostic factors, and patient outcome. Cancer Res.

[4] Portela A, Esteller M (2010). Epigenetic modifications and human disease. Nat Biotechnol.

[5] Seligson DB, Horvath S, McBrian MA, Mah V, Yu H, Tze S (2009). Global levels of histone modifications predict prognosis in different cancers. Am J Pathol.

[6] Seligson DB, Horvath S, Shi T, Yu H, Tze S, Grunstein M (2005). Global histone modification patterns predict risk of prostate cancer recurrence. Nature.

[7] Bauden M, Pamart D, Ansari D, Herzog M, Eccleston M, Micallef J (2015). Circulating nucleosomes as epigenetic biomarkers in pancreatic cancer. Clin Epigenet.

[8] Rahier JF, Druez A, Faugeras L, Martinet JP, Gehenot M, Josseaux E (2017). Circulating nucleosomes as new blood-based biomarkers for detection of colorectal cancer. Clin Epigenet.

[9] Shen H, Laird PW (2013). Interplay between the cancer genome and epigenome. Cell.

[10] Ganesan A, Arimondo PB, Rots MG, Jeronimo C, Berdasco M (2019). The timeline of epigenetic drug discovery: from reality to dreams. Clin Epigenet.

[11] Huang H, Lin S, Garcia BA, Zhao Y (2015). Quantitative proteomic analysis of histone modifications. Chem Rev.

[12] Colzani M, Noberini R, Romanenghi M, Colella G, Pasi M, Fancelli D (2014). Quantitative chemical proteomics identifies novel targets of the anti-cancer multi-kinase inhibitor E-3810. Mol Cell Proteom.

[13] Noberini R, Longuespee R, Richichi C, Pruneri G, Kriegsmann M, Pelicci G (2017). PAT-H-MS coupled with laser microdissection to study histone post-translational modifications in selected cell populations from pathology samples. Clin Epigenet.

[14] Noberini R, Restellini C, Savoia EO, Bonaldi T (2019). Enrichment of histones from patient samples for mass spectrometry-based analysis of post-translational modifications. Methods.

[15] Restellini C, Cuomo A, Lupia M, Giordano M, Bonaldi T, Noberini R (2019). Alternative digestion approaches improve histone modification mapping by mass spectrometry in clinical samples. Proteomics Clin Appl.

[16] Fowler CB, O'Leary TJ, Mason JT (2011). Protein mass spectrometry applications on FFPE tissue sections. Methods Mol Biol.

[17] Noberini R, Osti D, Miccolo C, Richichi C, Lupia M, Corleone G (2018). Extensive and systematic rewiring of histone post-translational modifications in cancer model systems. Nucleic Acids Res.

[18] Sanchini V, Bonizzi G, Disalvatore D, Monturano M, Pece S, Viale G (2016). A trust-based pact in research biobanks. from theory to practice. Bioethics.

[19] Noberini R, Uggetti A, Pruneri G, Minucci S, Bonaldi T (2016). Pathology tissue-quantitative mass spectrometry analysis to profile histone post-translational modification patterns in patient samples. Mol Cell Proteom.

[20] Noberini R, Bonaldi T (2017). A super-SILAC strategy for the accurate and multiplexed profiling of histone posttranslational modifications. Methods Enzymol.

[21] Maile TM, Izrael-Tomasevic A, Cheung T, Guler GD, Tindell C, Masselot A (2015). Mass spectrometric quantification of histone post-translational modifications by a hybrid chemical labeling method. Mol Cell Proteom.

[22] Ong SE, Mittler G, Mann M (2004). Identifying and quantifying in vivo methylation sites by heavy methyl SILAC. Nat Methods.

[23] Bremang M, Cuomo A, Agresta AM, Stugiewicz M, Spadotto V, Bonaldi T (2013). Mass spectrometry-based identification and characterisation of lysine and arginine methylation in the human proteome. Mol Biosyst.

[24] Yuan ZF, Sidoli S, Marchione DM, Simithy J, Janssen KA, Szurgot MR (2018). EpiProfile 2.0: a computational platform for processing epi-proteomics mass spectrometry data. J Proteome Res.

[25] Pesavento JJ, Mizzen CA, Kelleher NL (2006). Quantitative analysis of modified proteins and their positional isomers by tandem mass spectrometry: human histone H4. Anal Chem.

[26] Vizcaino JA, Deutsch EW, Wang R, Csordas A, Reisinger F, Rios D (2014). ProteomeXchange provides globally coordinated proteomics data submission and dissemination. Nat Biotechnol.

[27] Belov ME, Ellis SR, Dilillo M, Paine MRL, Danielson WF, Anderson GA (2017). Design and performance of a novel interface for combined matrix-assisted laser desorption ionization at elevated pressure and electrospray ionization with orbitrap mass spectrometry. Anal Chem.

[28] He L, Diedrich J, Chu YY, Yates JR (2015). Extracting accurate precursor information for tandem mass spectra by rawconverter. Anal Chem.

[29] Alexandrov T, Becker M, Deininger SO, Ernst G, Wehder L, Grasmair M (2010). Spatial segmentation of imaging mass spectrometry data with edge-preserving image denoising and clustering. J Proteome Res.

[30] Tyanova S, Temu T, Sinitcyn P, Carlson A, Hein MY, Geiger T (2016). The Perseus computational platform for comprehensive analysis of (prote)omics data. Nat Methods.

[31] Noberini R, Robusti G, Bonaldi T (2021). Mass spectrometry-based characterization of histones in clinical samples: applications, progresses, and challenges. FEBS J.

[32] Soldi M, Cuomo A, Bonaldi T (2014). Improved bottom-up strategy to efficiently separate hypermodified histone peptides through ultra-HPLC separation on a bench top Orbitrap instrument. Proteomics.

[33] Sidoli S, Bhanu NV, Karch KR, Wang X, Garcia BA (2016). Complete workflow for analysis of histone post-translational modifications using bottom-up mass spectrometry: from histone extraction to data analysis. J Vis Exp.

[34] Cornett DS, Reyzer ML, Chaurand P, Caprioli RM (2007). MALDI imaging mass spectrometry: molecular snapshots of biochemical systems. Nat Methods.

[35] Gutstein HB, Morris JS, Annangudi SP, Sweedler JV (2008). Microproteomics: analysis of protein diversity in small samples. Mass Spectrom Rev.

[36] Noberini R, Morales Torres C, Savoia EO, Brandini S, Jodice MG, Bertalot G (2020). Label-free mass spectrometry-based quantification of linker histone H1 variants in clinical samples. Int J Mol Sci.

[37] Kriegsmann J, Kriegsmann M, Casadonte R (2015). MALDI TOF imaging mass spectrometry in clinical pathology: a valuable tool for cancer diagnostics (review). Int J Oncol.

[38] Balluff B, Frese CK, Maier SK, Schone C, Kuster B, Schmitt M (2015). De novo discovery of phenotypic intratumour heterogeneity using imaging mass spectrometry. J Pathol.

[39] Inglese P, McKenzie JS, Mroz A, Kinross J, Veselkov K, Holmes E (2017). Deep learning and 3D-DESI imaging reveal the hidden metabolic heterogeneity of cancer. Chem Sci.

[40] Dilillo M, Ait-Belkacem R, Esteve C, Pellegrini D, Nicolardi S, Costa M (2017). Ultra-high mass resolution MALDI imaging mass spectrometry of proteins and metabolites in a mouse model of glioblastoma. Sci Rep.

[41] Gao G, Wang Z, Qu X, Zhang Z (2020). Prognostic value of tumor-infiltrating lymphocytes in patients with triple-negative breast cancer: a systematic review and meta-analysis. BMC Cancer.

