# Supplementary material for: Spatial epi-proteomics enabled by histone post-translational modification analysis from low-abundance clinical samples
Source: Clin Epigenetics. 2021 Jul 28;13:145. doi: 10.1186/s13148-021-01120-7 (PMC8317427; doi:10.1186/s13148-021-01120-7)
Supplement: Supplementary file 1 — Additional file 1. Supplementary tables and figures. [file 13148_2021_1120_MOESM1_ESM.docx]

**Noberini et al., Supplemental tables and figures**

**Figure S1**


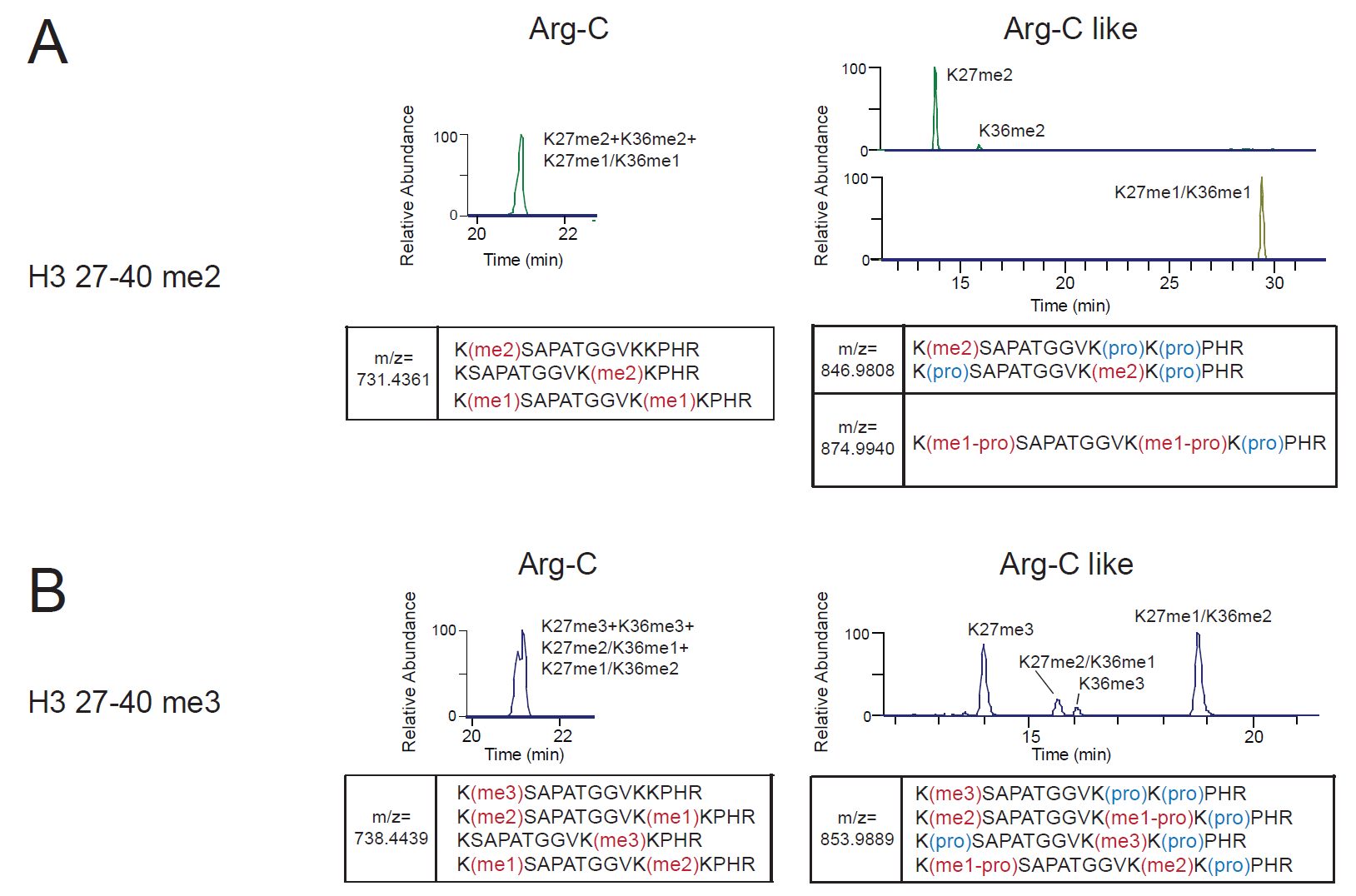


**Figure S1. Comparison of Arg-C and Arg-C-like digestions.** The Arg-C (left panels) and Arg-C-like (right panels) digestions generate peptides with the same sequence, but in Arg-C-like digested peptides unmodified and mono-methylated lysines are acylated (in this example with a propionil group). The presence of the acyl groups provides several advantages: 1) it causes differences in the m/z values for peptides that would have the same m/z with an Arg-C digestion (as shown in A for the H3 27-40 peptides containing either one dimethylation or two monomethylations); 2) it causes retention times shifts between isobaric peptides (as shown in A for H3K27me2/K36me2, and in B for H3K27me3/K36me3/K27me1K36me2/ K27me2K36me1).

**Figure S2**


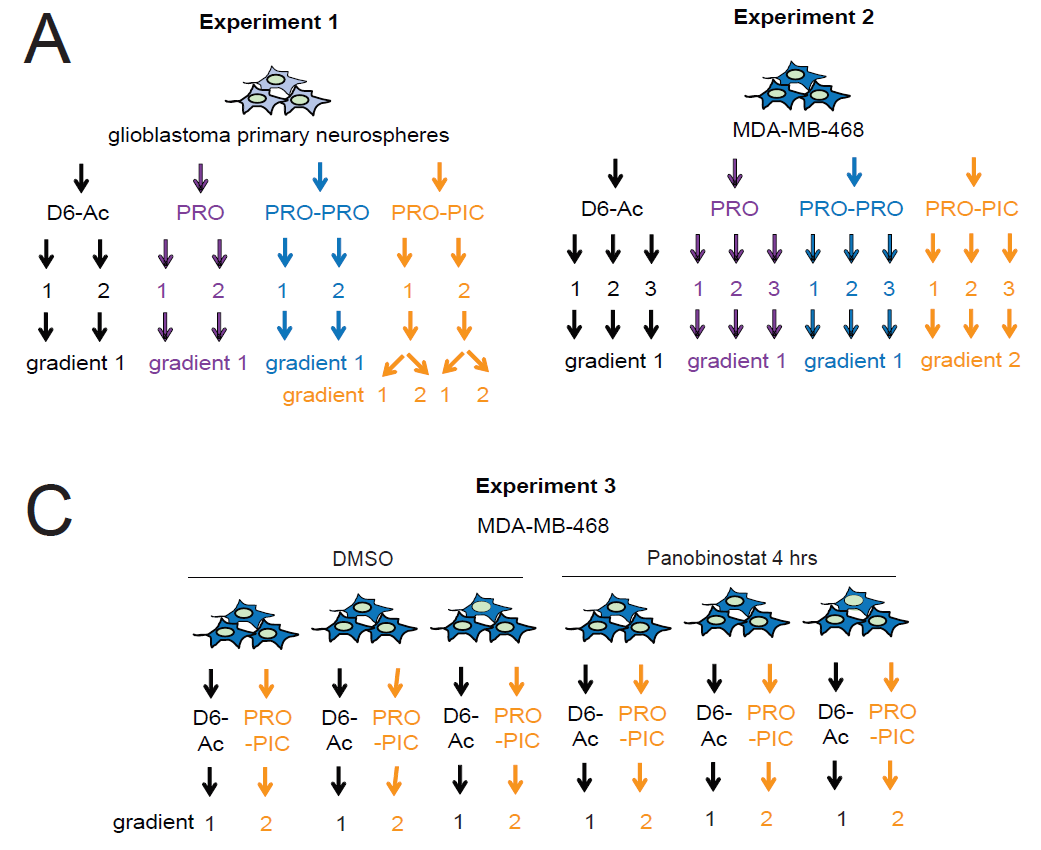


**Figure S2. Experimental design summary.** In order to compare different in-gel digestion methods, histone enriched/purified from two types of samples (glioblastoma primary neurospheres and the breast cancer cell line MDA-MS-468) were processed in parallel with the D3-Ac, PRO, PRO-PRO and PRO-PIC protocols. Neurospheres were processed in technical duplicates, using the same gradient (gradient 1, see Exp. Procedures and Figure S3) for all the samples; in addition, gradient 2 was also tested for the PRO-PIC samples. MDA-Mb-468 were processed in technical triplicates, using gradient 1 for the D3-Ac, PRO, PRO-PRO protocols, and gradient 2 for the PRO-PIC protocol.

**Figure S3**


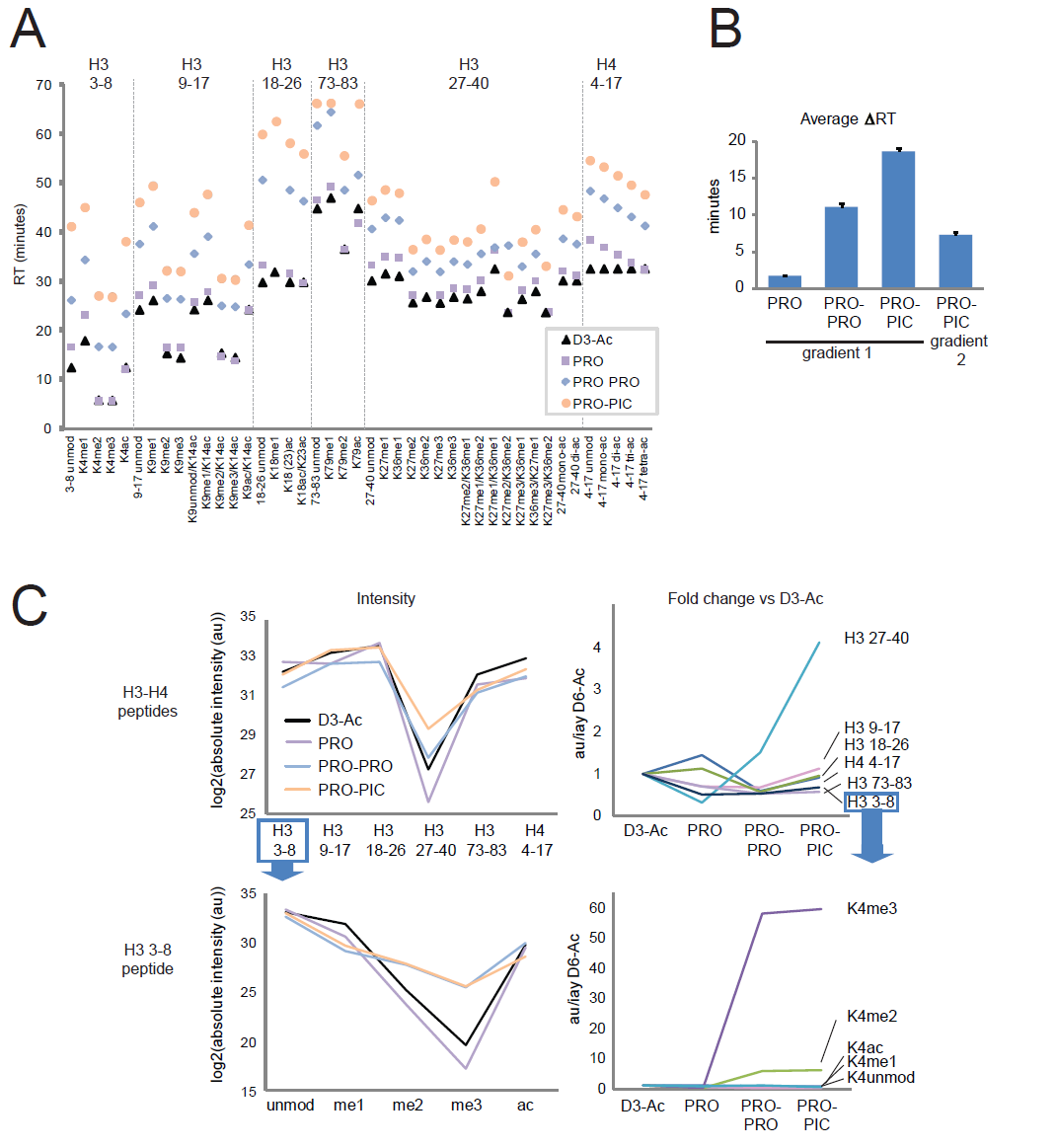


**Figure S3. Comparison of in-gel digestion methods.** (A) Reversed Phase Liquid Chromatography (RP-LC) retention times for the most common modified peptides detected in glioblastoma primary cells processed with the four in-gel digestion methods. The HPLC gradient was the same in all cases (gradient 1, see Figure S3). (B) Top panel: intensity (calculated as the sum of the intensities of all the differentially modified forms of a peptides) for six histone H3 and H4 peptides, obtained after digestion with the four in-gel digestion strategies. Bottom panel: intensity for the differentially modified forms of the H3 3-8 peptide. (C) The same data as in B is displayed as ratios compared with the D3-Ac protocol.

**Figure S4**


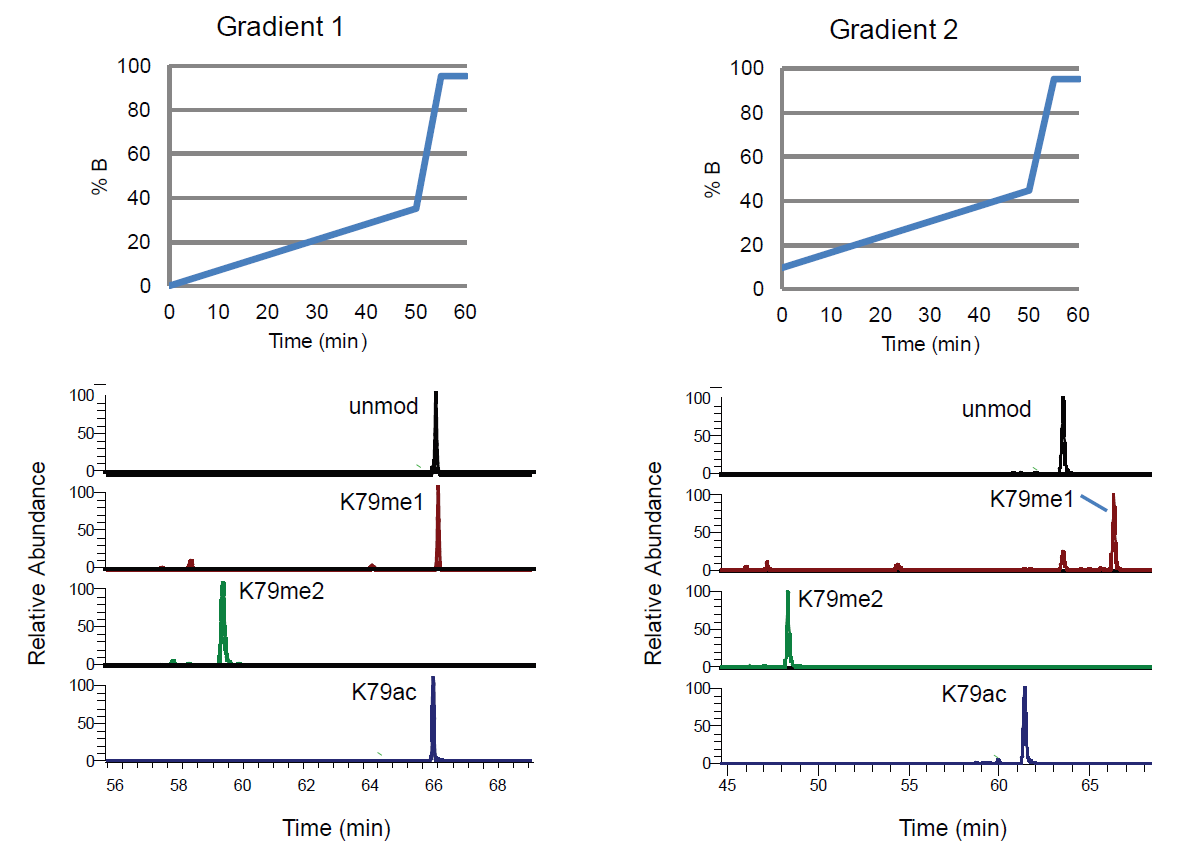


**Figure S4.** HPLC gradients and elution profiles of the differentially modified forms of the histone H3 peptide 73-83 obtained with the PRO-PIC digestion method, using two different gradients. Gradient 1: 50-min linear gradient of 0–35% solvent B; gradient 2: 50-min linear gradient of 10–45% solvent B.

**Figure S5**


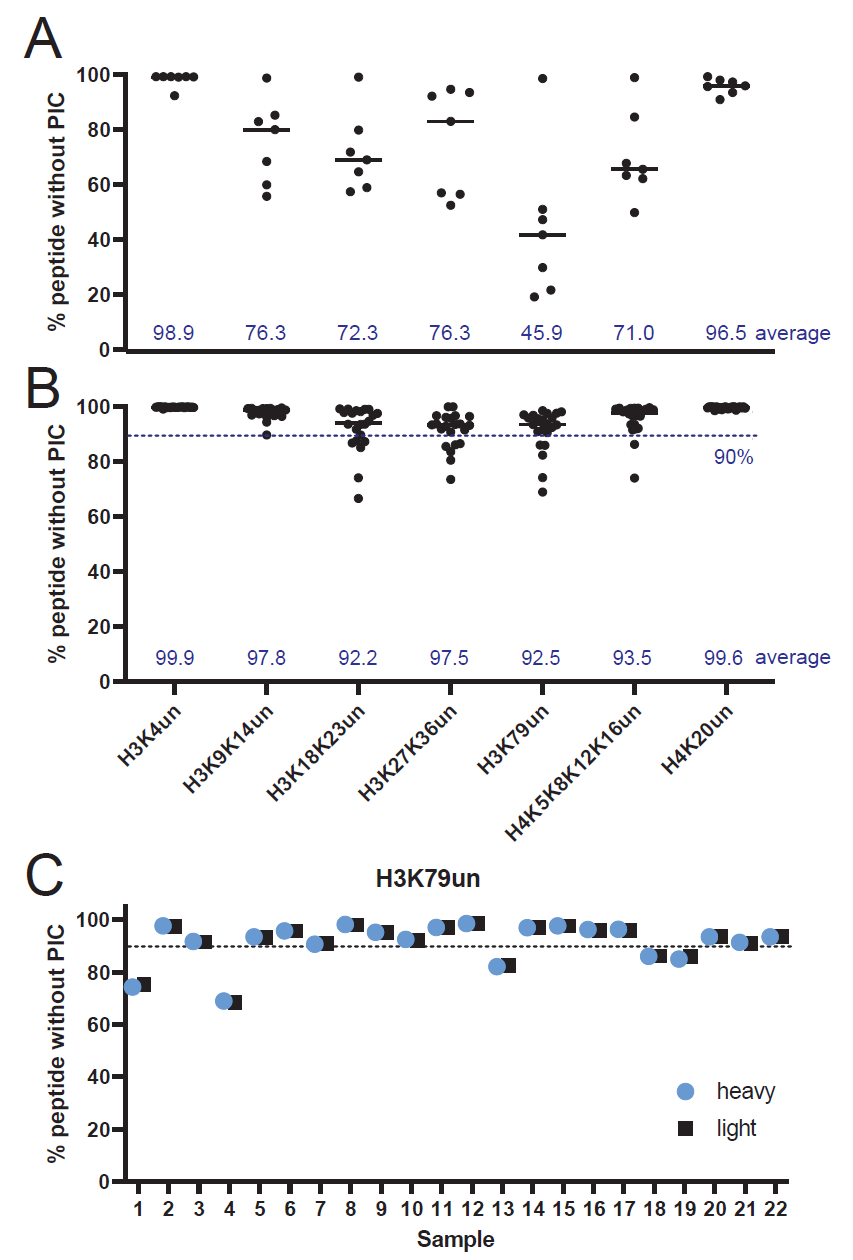


**Figure S5. Efficiency of N-terminal peptide derivatization with PIC. (A)** Percentage intensity of PIC-derivatized histone peptides by using the protocol published for in-solution digestions ((1)) (A) or our optimized protocol (with increased PIC amount and incubation time) (B). The percentage values were calculated by dividing the area under the curve (AUC) obtained for PIC-derivatized peptides for the sum of PIC-derivatized and not derivatized peptides. Only the unmodified forms of histone H3 and H4 most common peptides were analyzed. The values change substantially among peptides (e.g. H3K79unmod is generally less derivatized than the other peptides) and among samples, but are substantially decreased when using the optimized protocol. n=7 in A, and n=22 in B. **(C)** Percentage intensity of PIC-derivatized histone H3K79unmod peptide using the optimized PRO-PIC protocol in 22 samples. Although there is variability among samples, the values are almost identical for the light and heavy channel, which were acquired together, indicating that even if a certain degree of technical variability exists, a lower derivatization yield does not affect peptide quantitation when using a SILAC spike-in set up.

**Figure S6**


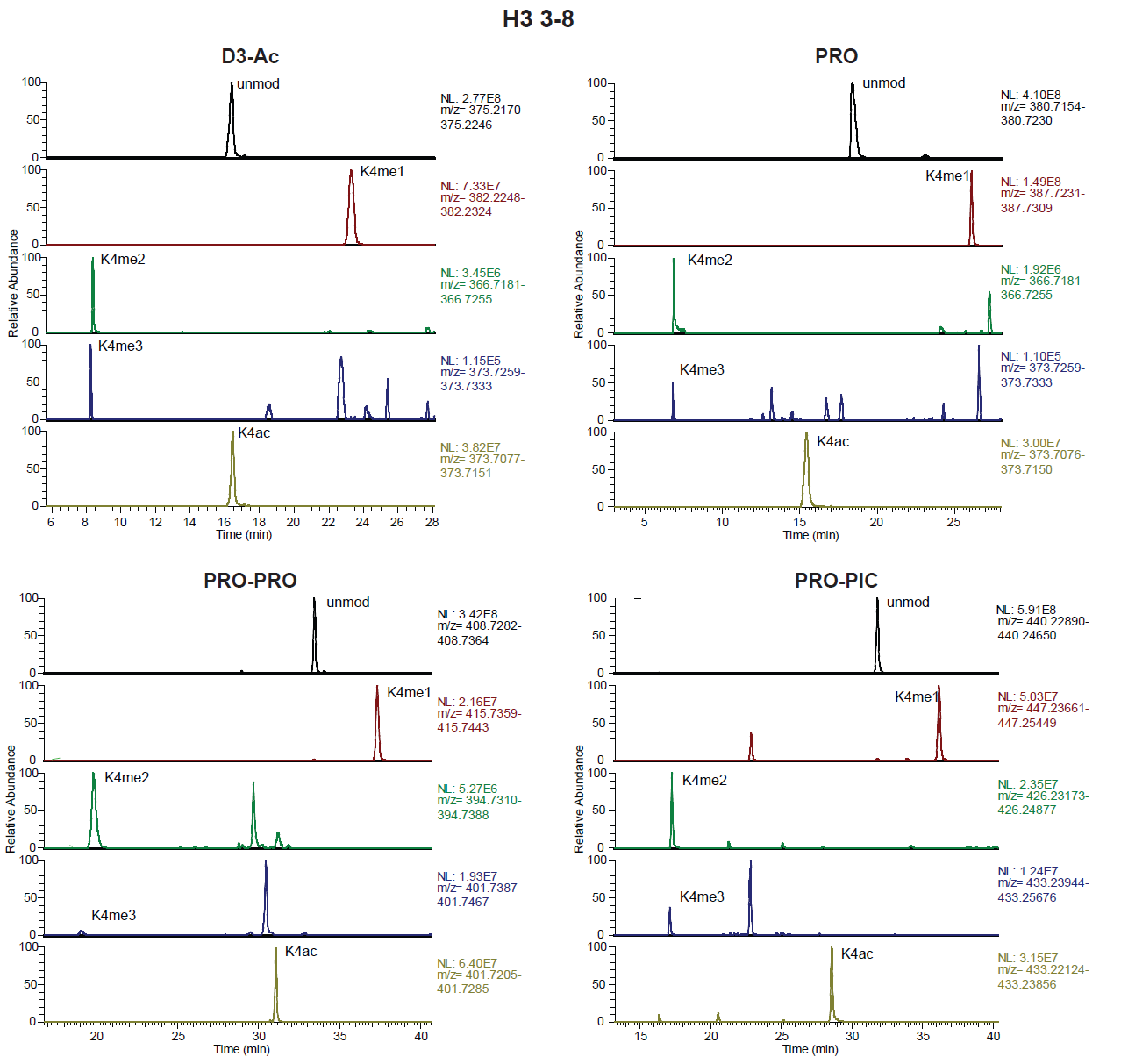


**Figure S6.** Representative elution profiles of the differentially modified forms of the histone H3 peptide 3-8 obtained with the indicated in-gel digestion methods.

**Figure S7**


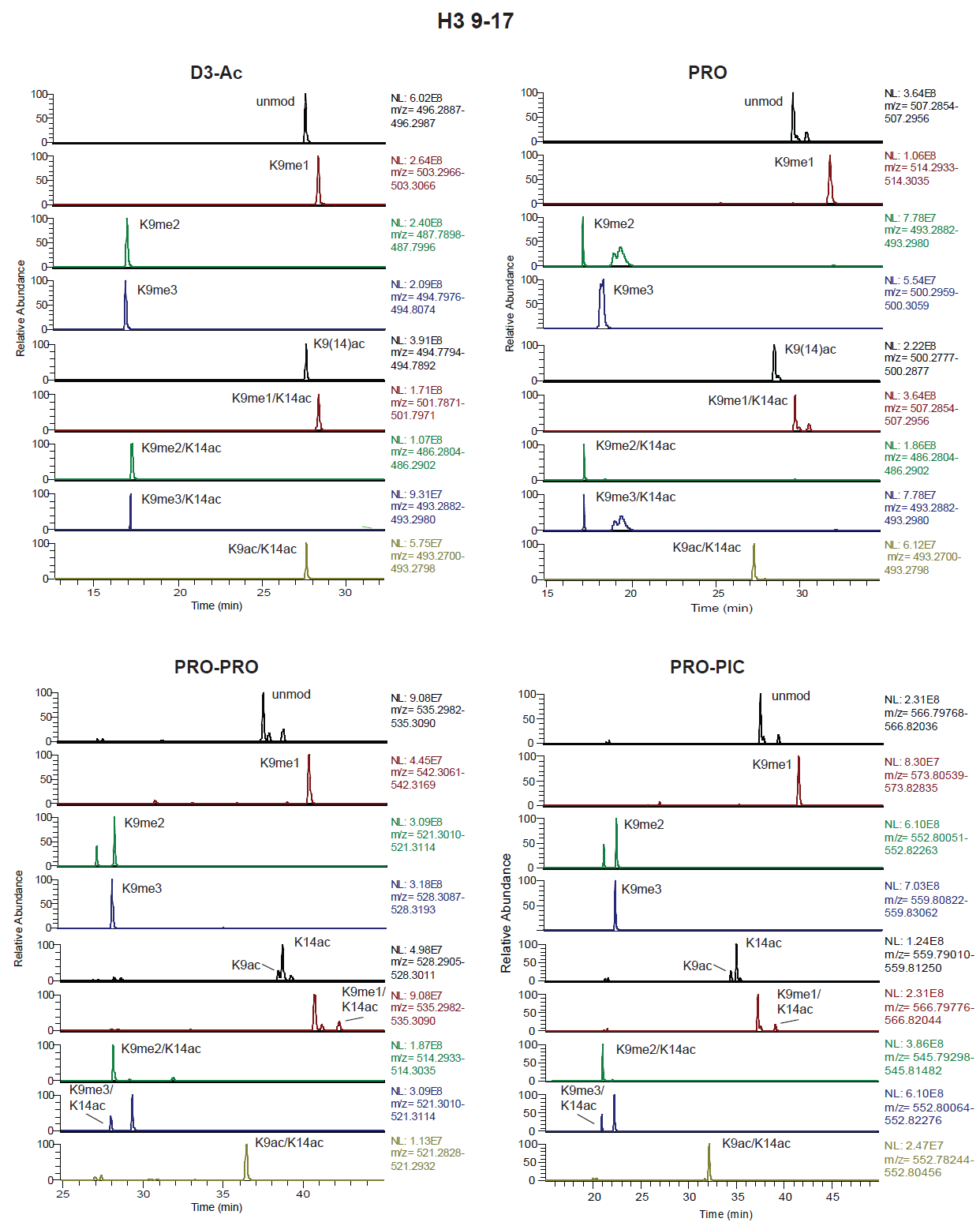


**Figure S7.** Representative elution profiles of the differentially modified forms of the histone H3 peptide 9-17 obtained with the indicated in-gel digestion methods.

**Figure S8**


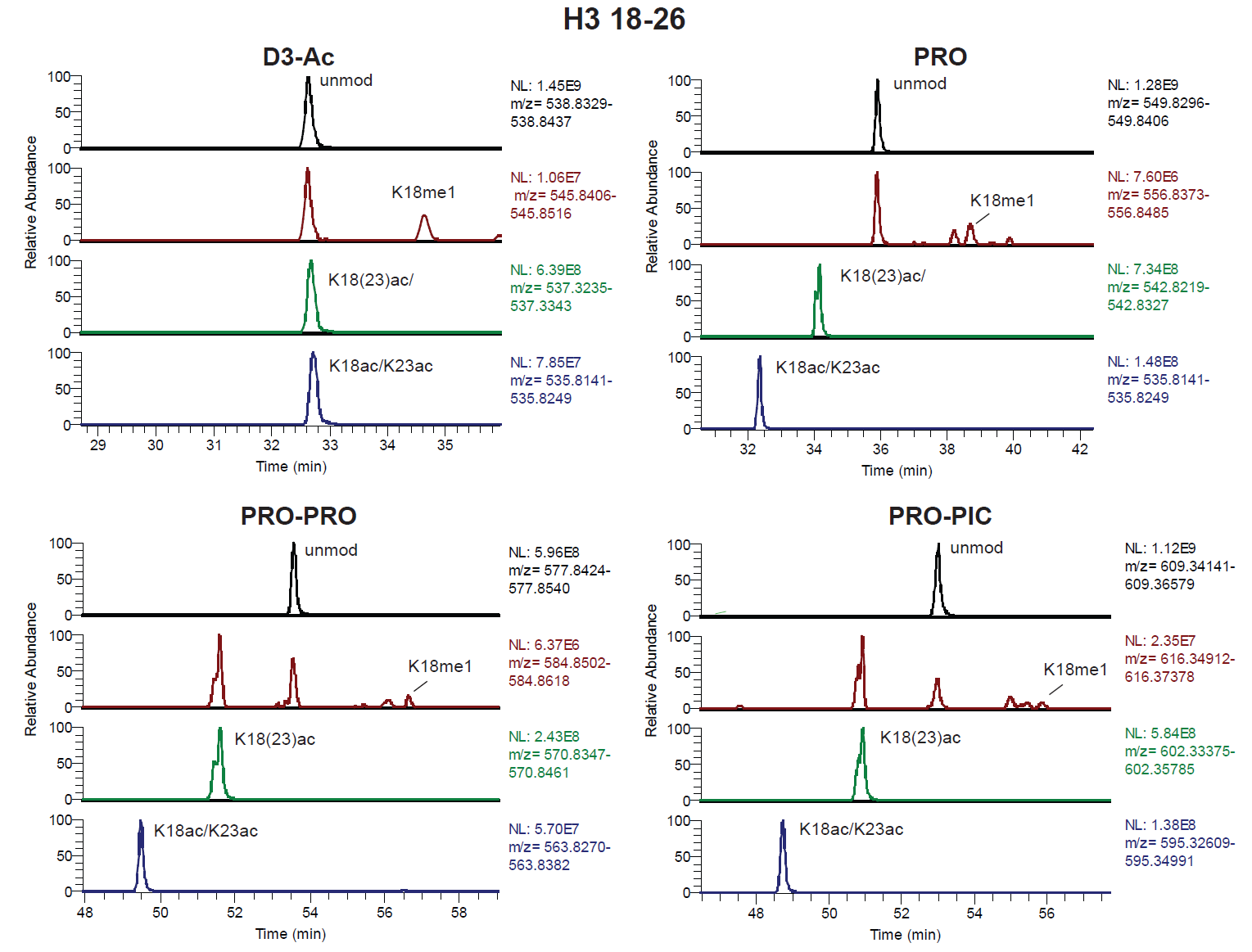


**Figure S8.** Representative elution profiles of the differentially modified forms of the histone H3 peptide 18-26 obtained with the indicated in-gel digestion methods.

**Figure S9**


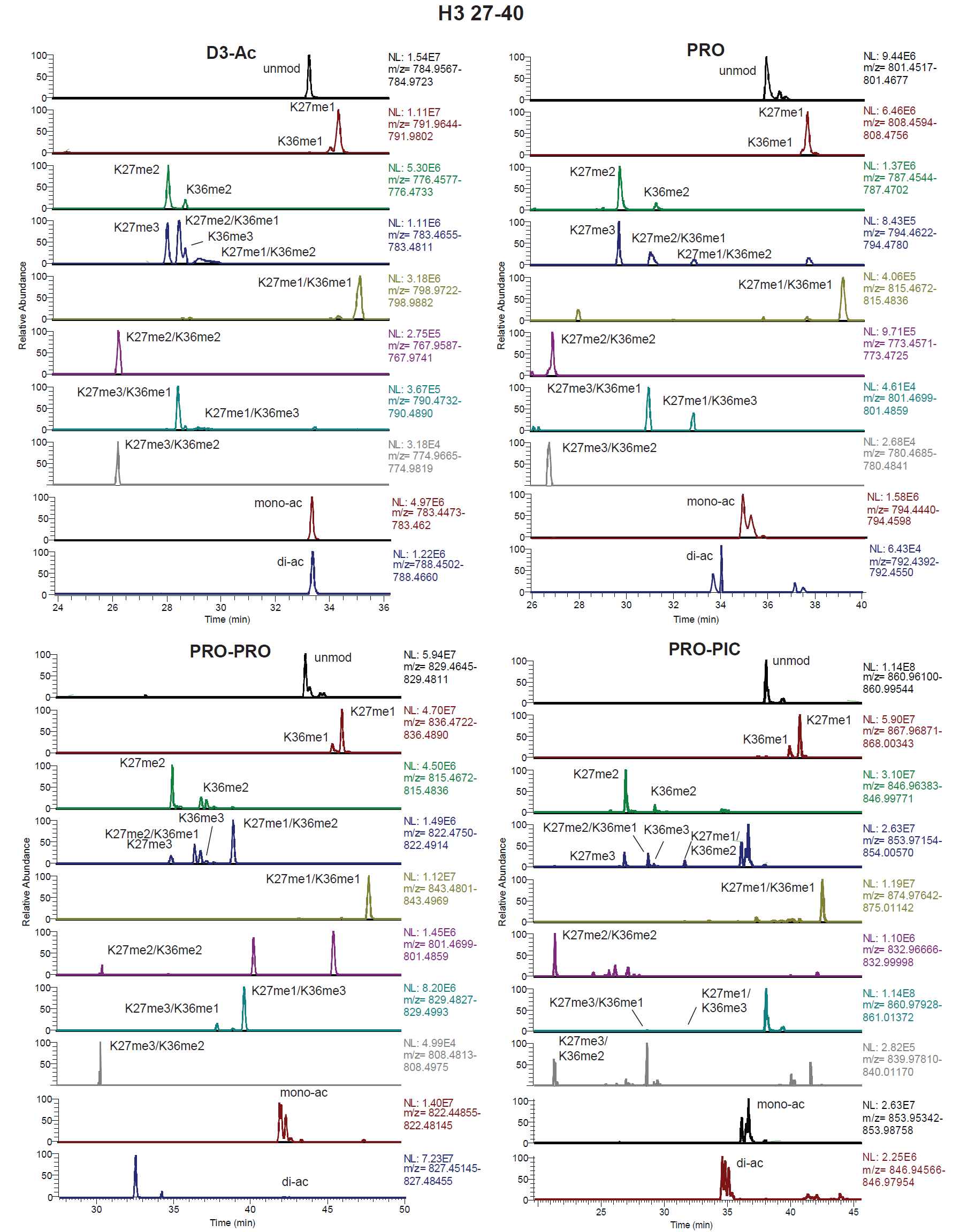


**Figure S9.** Representative elution profiles of the differentially modified forms of the histone H3 peptide 27-40 obtained with the indicated in-gel digestion methods.

**Figure S10**


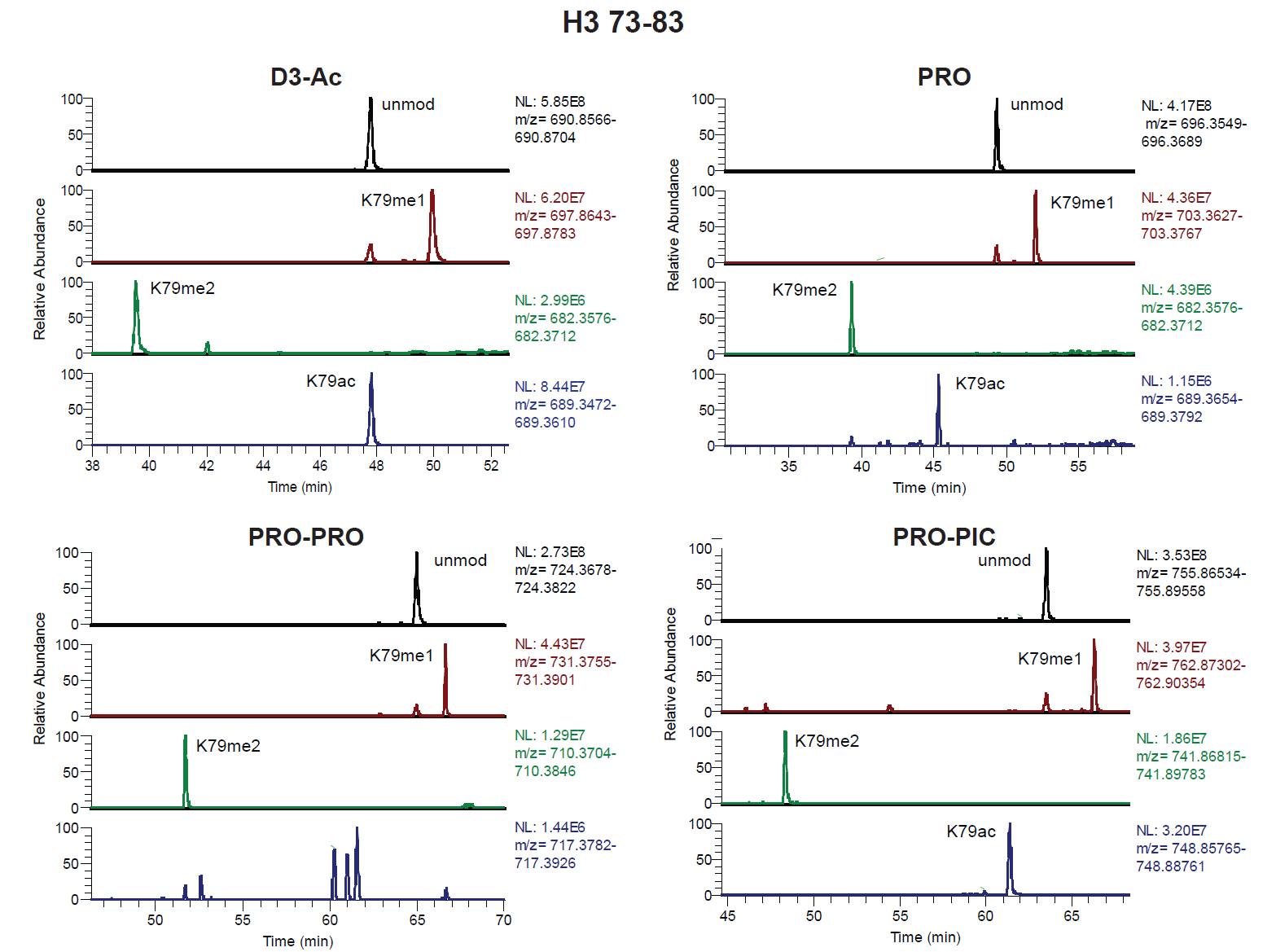


**Figure S10.** Representative elution profiles of the differentially modified forms of the histone H3 peptide 73-83 obtained with the indicated in-gel digestion methods.

**Figure S11**


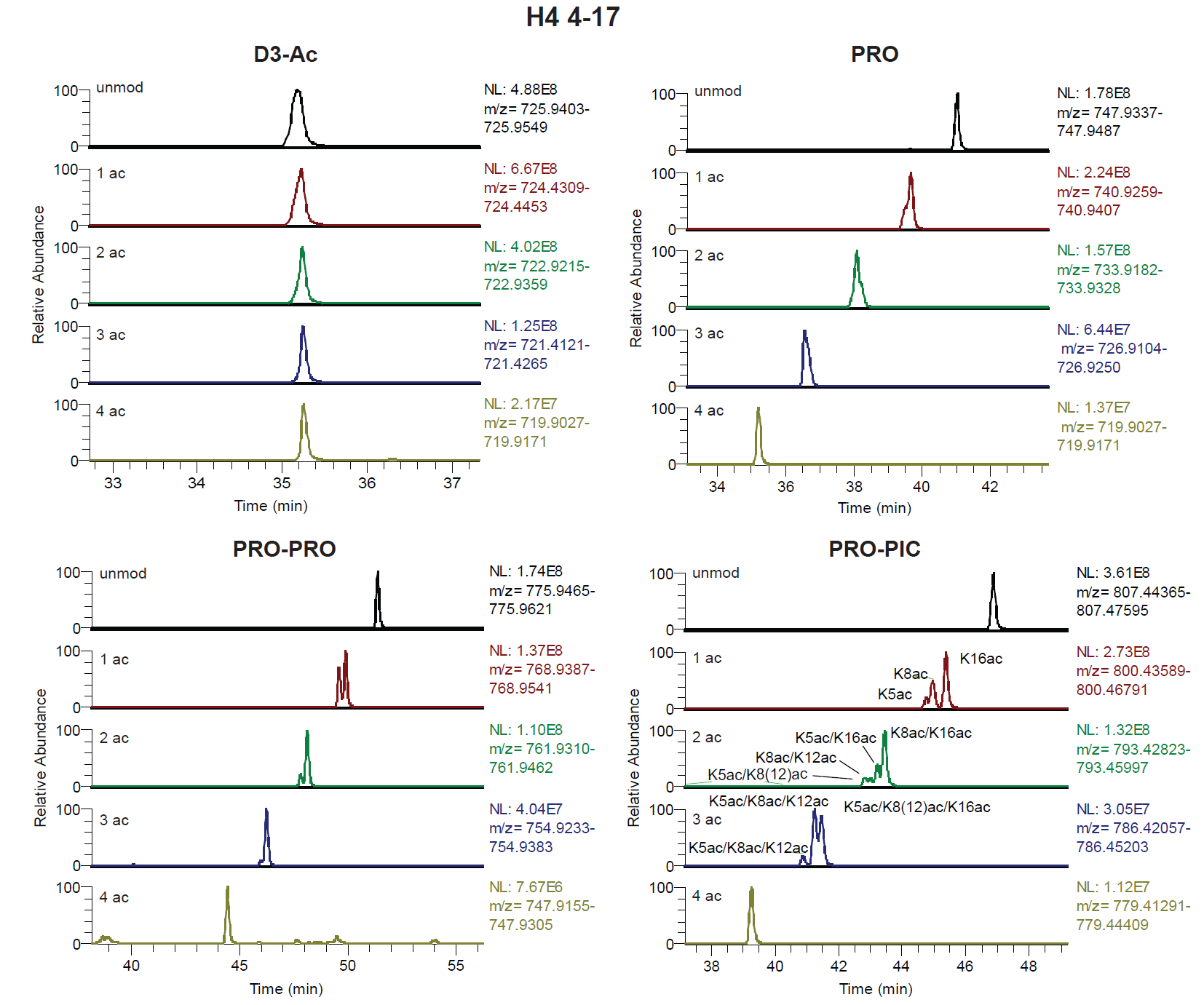


**Figure S11.** Representative elution profiles of the differentially modified forms of the histone H4 peptide 4-17 obtained with the indicated in-gel digestion methods.

**Figure S12**


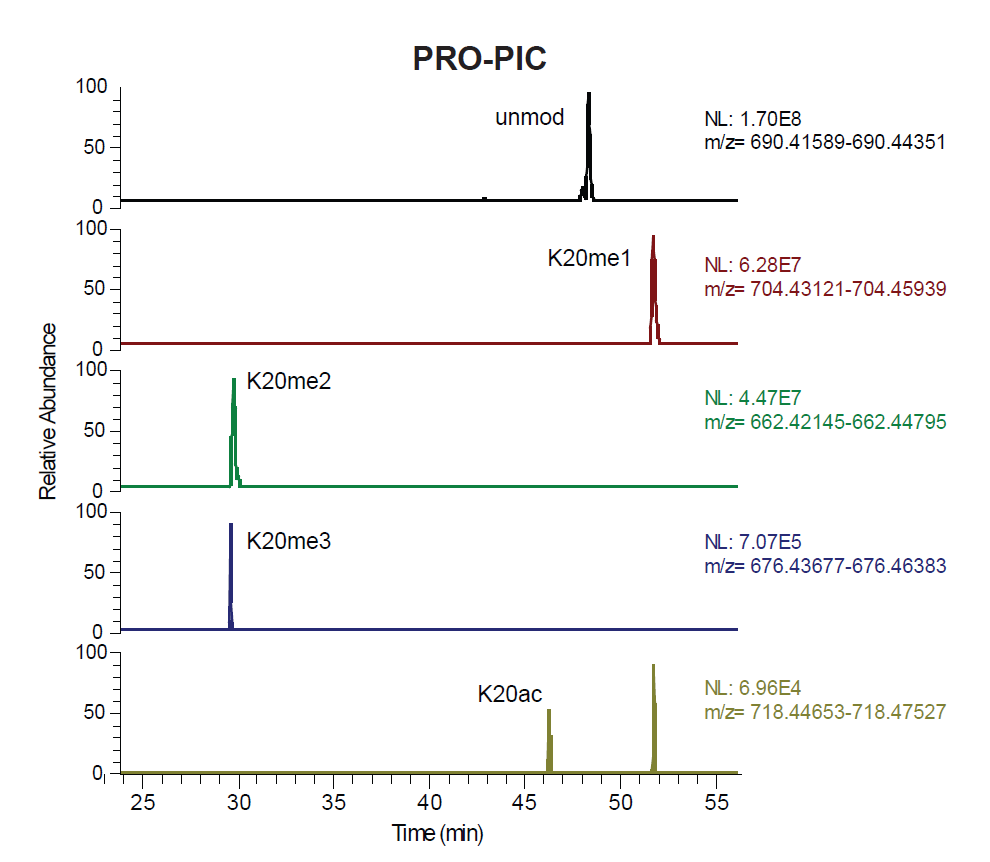


**Figure S12.** Representative elution profiles of the differentially modified forms of the histone H4 peptide 20-23 obtained with the PRO-PIC in-gel digestion method.

**Figure S13**


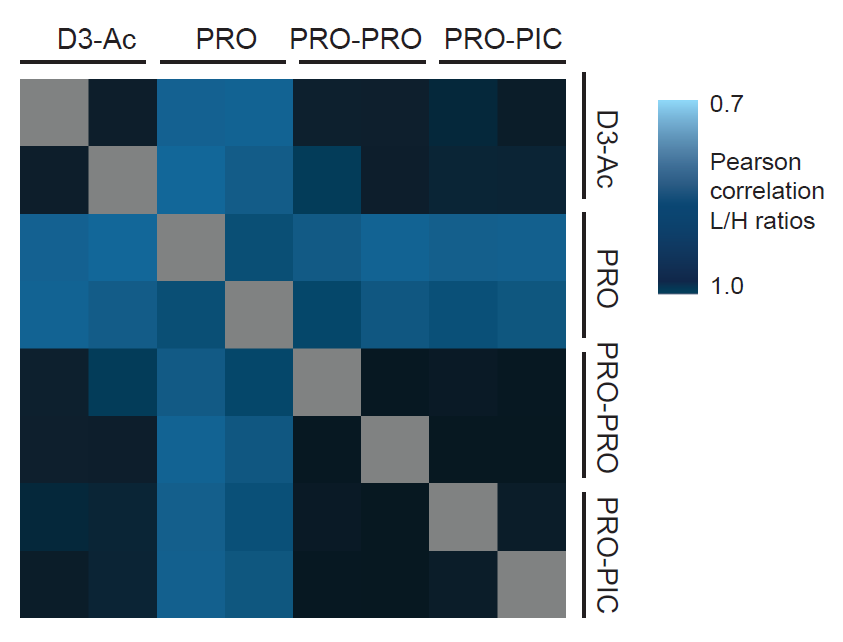


**Figure S13.** Correlation matrix based on Pearson correlation coefficients of L/H ratios (light channel: glioblastoma neurospheres; heavy channel: super-SILAC spike-in) for histone PTMs quantified from samples processed in technical duplicates through the four in-gel digestion protocols.

**Figure S14**


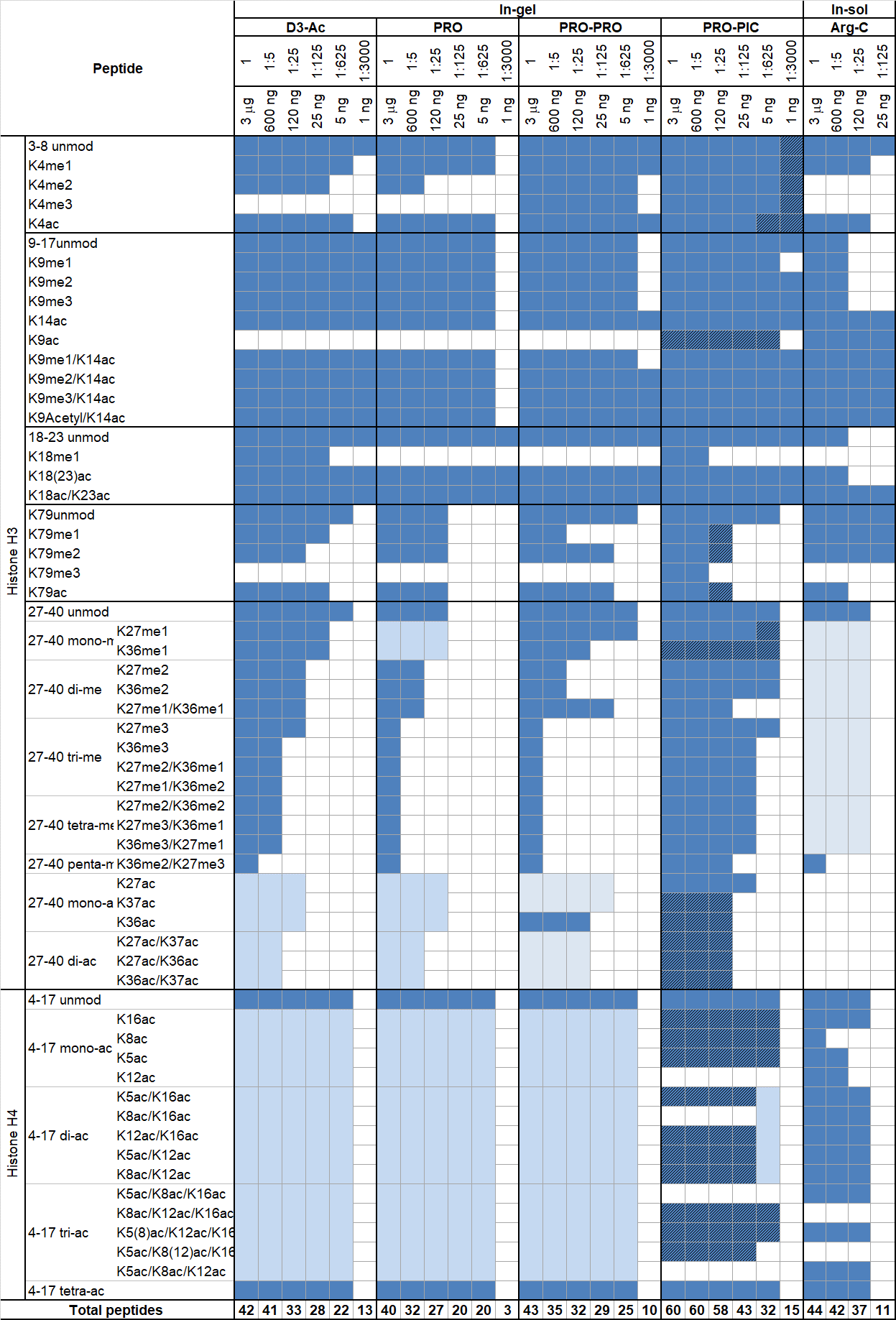


**Figure S14 (previous page).** List of peptides identified and quantified using the four in-gel or the Arg-C in-solution digestion protocols. The lighter blue color indicates isobaric peptides that could not be quantified individually, darker cells indicate peptides that could not be quantified by the EpiProfile 2.0 Software, but were quantified manually.

**Supplementary references**

1. Maile, T.M., Izrael-Tomasevic, A., Cheung, T., Guler, G.D., Tindell, C., Masselot, A., Liang, J., Zhao, F., Trojer, P., Classon, M. *et al.* (2015) Mass spectrometric quantification of histone post-translational modifications by a hybrid chemical labeling method. *Mol. Cell. Proteomics*, **14**, 1148-1158.
